# An Integrated Performance-Based Screening Framework for Fiber-Reinforced Concrete Floating Dock Blocks for Offshore Renewable Energy Applications

**DOI:** 10.3390/ma19214533

**Published:** 2026-10-28

**Authors:** Kasra Hosseinmostofi, Fatemeh Soltanzadeh, Ali Edalat-Behbahani, Isabel B. Valente, Eduardo N. B. Pereira

**Affiliations:** 1 Institute for Sustainability and Innovation in Structural Engineering, Department of Civil Engineering, School of Engineering, University of Minho, 4800-058 Guimarães, Portugal; 2 DIS/CREADIS, Ørstedsvej 10, Stilling, DK-8660 Skanderborg, Denmark

**Keywords:** floating dock blocks, offshore renewable energy, fiber-reinforced self-compacting concrete, recycled steel fibers, hybrid steel fibers, post-cracking response, fiber orientation, FRP–concrete bond, impact resistance, life-cycle assessment, performance-based screening

## Abstract

Selecting fiber-reinforced concrete (FRC) for thin marine shells is a multi-constraint decision because lower cradle-to-gate global warming potential (GWP) at equal volume may be offset by post-cracking requirements, interface behavior, impact response, or detailing. This study introduces a performance-constrained, multi-evidence screening framework and illustrates it with traceable mechanical, fiber-field, marine-bond, impact, and life-cycle datasets for four self-compacting concrete systems with 90 kg/m^3^ of industrial and/or recycled steel fibers. Unlike a compensatory environmental–mechanical index, the framework applies simultaneous normalized response constraints, independent interface and component gates, deterministic ranking-sensitivity analysis, and component carbon-parity budgets. Compressive strengths were 60.03–66.10 MPa, whereas post-cracking properties varied substantially. At 80% retained performance, beam work favors I30R60, whereas an additional 3 mm splitting constraint favors I45R45. For a 0.144 m^3^ panel, I45R45 has 3.89 kg CO_2_-eq less reported cradle-to-gate GWP than I90R0, corresponding to only 5.50% additional concrete before carbon parity under the source inventory. Results depend on published means and inventory assumptions. The four documented 4.0 m impact tests (one slab per tested composition) illustrate the component gate as a proof of concept rather than a statistically supported pass/fail criterion. Replicated component testing and project-specific verification are required before design qualification.

## 1. Introduction

Prefabricated floating dock shells are exposed to service loading, handling and transport actions, aggressive marine environments, and accidental impact. Their relatively thin sections also impose stringent requirements on concrete flow, fiber dispersion and orientation, crack bridging, and reinforcement anchorage. Consequently, compressive strength alone is insufficient for material screening; the selected composite must retain adequate matrix, interface, and component-level performance throughout the relevant exposure and loading conditions.

The doctoral research underlying this study addresses a modular floating system comprising an external fiber-reinforced self-compacting concrete (FRSCC) shell and a lightweight core produced from cork residues [1]. Industrial steel fibers (ISFs), tire-derived recycled steel fibers (RSFs), and ISF–RSF hybrids were investigated as distributed reinforcement, while fiber-reinforced polymer (FRP) bars were evaluated for interfaces exposed to seawater. The intended applications include interconnected floating blocks and support infrastructure for offshore renewable-energy systems. The available experiments characterize material, interface, and component responses; full-system buoyancy, stability, watertightness, and connection behavior remain outside the present experimental scope.

These source studies address distinct domain-specific questions, whereas the previous environmental–mechanical assessment [2,3,4,5] employed a compensatory index that divides normalized beam work by a weighted environmental score. Such an index can improve when the environmental benefit offsets the loss of a mechanically indispensable property, and it does not incorporate crack-opening-specific exclusion limits or changes in component geometry. The original contribution here is an explicit decision protocol: (i) simultaneous mechanical-admissibility constraints at prescribed crack-opening states; (ii) deterministic ranking-sensitivity limits; (iii) continuous boundaries identifying the lowest-GWP candidate within the admissible set; and (iv) component carbon-parity allowances for added concrete and local thickening. The fiber-field, conditioned-bond, and single-impact observations are independent evidence gates, never compensating numerical contributions to the environmental score. These are new engineering decisions derived from published inputs, not additional experimental campaigns or independently validated material properties.

The importance of linking manufacturing conditions to mechanical qualifications is well established. Faustmann et al. [6] showed that specimen production and wall effects influence the direct tensile response of steel-fiber-reinforced concrete, while hosseinmostofi et al. [7] related fiber distribution and orientation in full-scale walls to concrete consistency and casting procedure. These studies support representative sampling from component-scale castings and direction-specific mechanical verification; however, they do not provide a universal orientation correction applicable to the mixtures considered here.

Performance-based FRC design already recognizes residual response as a key link between material characterization and structural use [8]. Research on recycled steel fibers further demonstrates that performance depends strongly on the recovery process, geometry, surface condition, and hybridization. Aiello et al. [9] reported effective pull-out and post-cracking behavior for tire-derived fibers. Caggiano et al. and hosseinmostofi et al. [3,4,10] showed that suitably characterized recycled fibers can replace manufactured fibers without a major mechanical penalty, and Hu et al. [11,12] demonstrated the potential of blended fibers and longer recycled tire steel cords. Accordingly, fiber origin alone cannot define a reliable performance ranking. Reviews of steel-fiber SCC and recycled-fiber concrete similarly emphasize the coupled influence of production, post-cracking response, and environmental burden [13,14].

The central hypothesis is that material selection for a floating concrete shell cannot be determined from environmental burden at equal volume alone: the preferred fiber system depends on the crack-opening state that must be sustained and on the component, detailing required to satisfy interface and impact demands. The study therefore addresses three linked questions: (i) which post-cracking requirement governs the mechanically admissible set; (ii) how sensitive is the resulting material ranking to the reported mean values; and (iii) how much additional concrete or local thickening can be introduced before the material-level carbon advantage is eliminated? The analysis derives joint screening limits, deterministic ranking sensitivities, continuous selection boundaries, and component-scale carbon budgets from internally compatible data. Fiber-field, marine-bond, and impact evidence remain separate qualification gates. This non-compensatory architecture solves a cross-scale engineering decision problem that is not addressed by any of the individual source studies. Finite-element simulations, constitutive-model calibration, and simulation-derived results are deliberately excluded.

## 2. Evidence Provenance, Data Compatibility, and Original Analysis

### 2.1. Evidence Provenance and Compatibility

Table 1 establishes the provenance, compatibility, and permitted analytical role of each evidence stream. The doctoral record provides the complete experimental context, while the cited papers provide traceable mechanical, imaging, interface, and environmental measurements. The present study does not relabel source experiments as newly performed. Instead, it treats them as experimental inputs to newly formulated cross-scale analyses. Direct numerical integration is permitted only where the mechanical and environmental values belong to the same internally paired benchmark family; evidence from other programs is used as an independent qualification gate when mixture matrix, batch, age, geometry, or exposure differs. This rule prevents apparent agreement across similarly named fiber systems from being mistaken for batch-level equivalence.

The source experiments therefore function as traceable inputs, whereas the primary analytical outputs of this study are the multi-constraint admissibility formulation in Equations (9) and (10), the ranking-sensitivity boundary in Equation (11), the component carbon-parity formulation in Equations (12) and (13), the decision regions summarized in Section 8, and the derivative component quantities in same section. These cross-scale outputs were not reported in the individual source studies [2,3,4,5]. The Article consequently contributes a new methodology and new derivative data while maintaining explicit provenance of every experimental input.

The four reinforced systems are designated I90R0, I45R45, I30R60, and I0R90, where I and R denote the respective ISF and RSF dosages in kg/m^3^. The impact series additionally included plain SCC and did not include I30R60. A common mixture label identifies the fiber combination, but not necessarily the same concrete batch or matrix across every experimental program. For example, the thesis reference mixture contained 472 kg/m^3^ of cement, whereas the 2022 LCA inventory used 500 kg/m^3^; coarse-aggregate contents also differed. The LCA and its paired mechanical dataset are therefore treated as one internally consistent benchmark family, and their environmental burdens are not assigned to the later impact slabs as batch-specific footprints.

Data integration requires explicit control of specimen geometry and the statistical unit. The modified-splitting reports describe nominal panels of 570 × 570 × 240 mm, approximately 150 mm diameter cores cut into nominal 60 mm disks, and notches defining a reduced load-transfer section [3]. The source calculation specifies an effective stress diameter of 120 mm and an effective ligament thickness of 50 mm, rather than simply using the nominal disk dimensions [3]. However, the source descriptions differ on the number of disks (including totals of 60 and 96) and the correspondence between extraction counts and direction-specific test groups. It is therefore not defensible to infer a uniform effective replicate count, a single independent panel-level sample size, or a reconstructed characteristic strength. Published 1 and 3 mm mean nominal stresses and their reported coefficients of variation are retained as within-source exploratory indicators [2]; their direction/depth-specific uncertainty cannot be recovered without specimen-level records. The impact series described for this demonstration comprises four slabs, one per tested mixture, with I30R60 absent [1,5].

### 2.2. Screening Sequence and Interpretation of Response Quantities

The proposed framework follows seven sequential, but feedback-enabled, decisions: define component-level requirements; identify candidate materials; qualify mixing and casting; establish mechanical and fiber-field responses; verify the reinforcement interface after relevant conditioning; assess component behavior; and compare environmental burdens only among candidates that satisfy the preceding requirements. The sequence is intentionally iterative. For example, an anchorage requirement may increase local thickness or reinforcement demand and thereby require the environmental comparison to be repeated with the revised quantities.

The formulation is intentionally separated into three levels of decision. First, candidate mixtures are screened against explicit mechanical requirements, expressed either as project-specific absolute limits or, for the present case-study demonstration, as normalized retained-response ratios relative to I90R0. Second, mandatory interface and component requirements are treated as non-compensatory gates: a favorable environmental result cannot offset a failed or untested mandatory property. Third, environmental preference is evaluated only within the surviving set and is recalculated if detailing changes material quantities. This architecture differs from a single weighted eco-efficiency score because failure of a prescribed performance constraint cannot be compensated by improvement in another criterion. Section 8 provides mathematical implementation and the case-study decision boundaries.

Measured responses, derived indicators, and design recommendations are kept distinct throughout the framework. Mean cylinder strength is not a characteristic design strength; nominal modified-splitting stress is not a direct tensile strength; beam work is not equivalent to collision-energy capacity of a slab; and average pull-out bond stress from a cube is not a universal anchorage resistance. A laboratory result supports only the tested material, geometry, exposure, and loading domain. Evidence that is absent from a required property remains an explicit qualification gap rather than an implicit pass.

The joint quantitative screen uses only the mechanically and environmentally paired dataset reported in [2]. Each mixture mean is normalized to I90R0 and evaluated using beam work together with modified-splitting stresses at 1 and 3 mm crack openings. These indicators represent integrated flexural response and two distinct stages of crack bridging and are therefore retained as separate constraints. A common retained-performance fraction is introduced only to explore sensitivity of the shortlist; the formulation also permits different thresholds for each indicator. No value used in the illustrative screen is proposed as a marine design limit.

Within the mechanically admissible set, candidate selection initially minimizes cradle-to-gate GWP. A deterministic perturbation analysis then quantifies the equal and opposing fractional changes in competing means required to reverse their ranking; this is a sensitivity measure, not a probability or confidence interval. Compatible per-volume GWP values are subsequently mapped to a common panel geometry to determine carbon allowances for additional concrete volume and local thickening, while non-concrete component burdens are introduced explicitly. Interface and impact results are used as subsequent qualification evidence and are not merged into the paired LCA dataset. No response from an incompatible batch family is used to improve another mixture’s environmental or structural ranking. The framework is demonstrated using the datasets from which it was constructed and therefore requires prospective validation on independently produced components.

## 3. Constituent Materials, Mixture Design, and Production

### 3.1. Constituent Characterization

The mixtures were developed from previously investigated fiber-reinforced selfcompacting concrete (FRSCC) formulations [2,3,4,5]. Ordinary Portland cement, CEM I 52.5 R

(CEM I 52.5 R; Secil, Porto, Portugal), fine and coarse river sands (maximum sizes 2.4 and 4.8 mm, Unibetão, Braga, Portugal), crushed granite (12.5 mm, Unibetão, Braga, Portugal), Class F fly ash, and limestone filler (both from Sameca Produtos Químicos S.A., Porto, Portugal)., a polycarboxylate-ether superplasticiser (Glenium SKY 617; Master Builders Solutions, Prior Velho, Portugal) and tap water were used. Hooked-end ISFs were supplied by Spaji’c d.o.o. (Negotin, Serbia) and tyre-derived RSFs were sourced from RETIRE S.A. (Drama, Greece), [1,2,3,4,5].

Reported fineness moduli were 1.913, 3.84, and 7.00 for fine sand, coarse sand, and granite, respectively, with corresponding water absorptions of 10.64%, 5.08%, and 1.58% [1]. Because aggregate moisture directly affects effective mixing water, production qualification should record grading, absorption, and moisture corrections using identified procedures such as ASTM C136/C136M, C127, and C128 [15,16,17]. These standards define appropriate control measurements for future production; the reported thesis values remain source-study test results.

The ISFs were hooked-end fibers with a nominal length of 35 mm and diameter of 0.55 mm, corresponding to an aspect ratio of approximately 64. The RSFs had reported a mean length and diameter of 33 mm and 0.38 mm, with coefficients of variation (CoVs) of 38% and 40%, respectively [2,3]. Their broad dimensional distribution and irregular geometry are better represented by measured length and diameter distributions than by a single nominal aspect ratio; notably, the mean of individual aspect ratios is not necessarily equal to the ratio of the mean length to the mean diameter.

**Figure 1:**
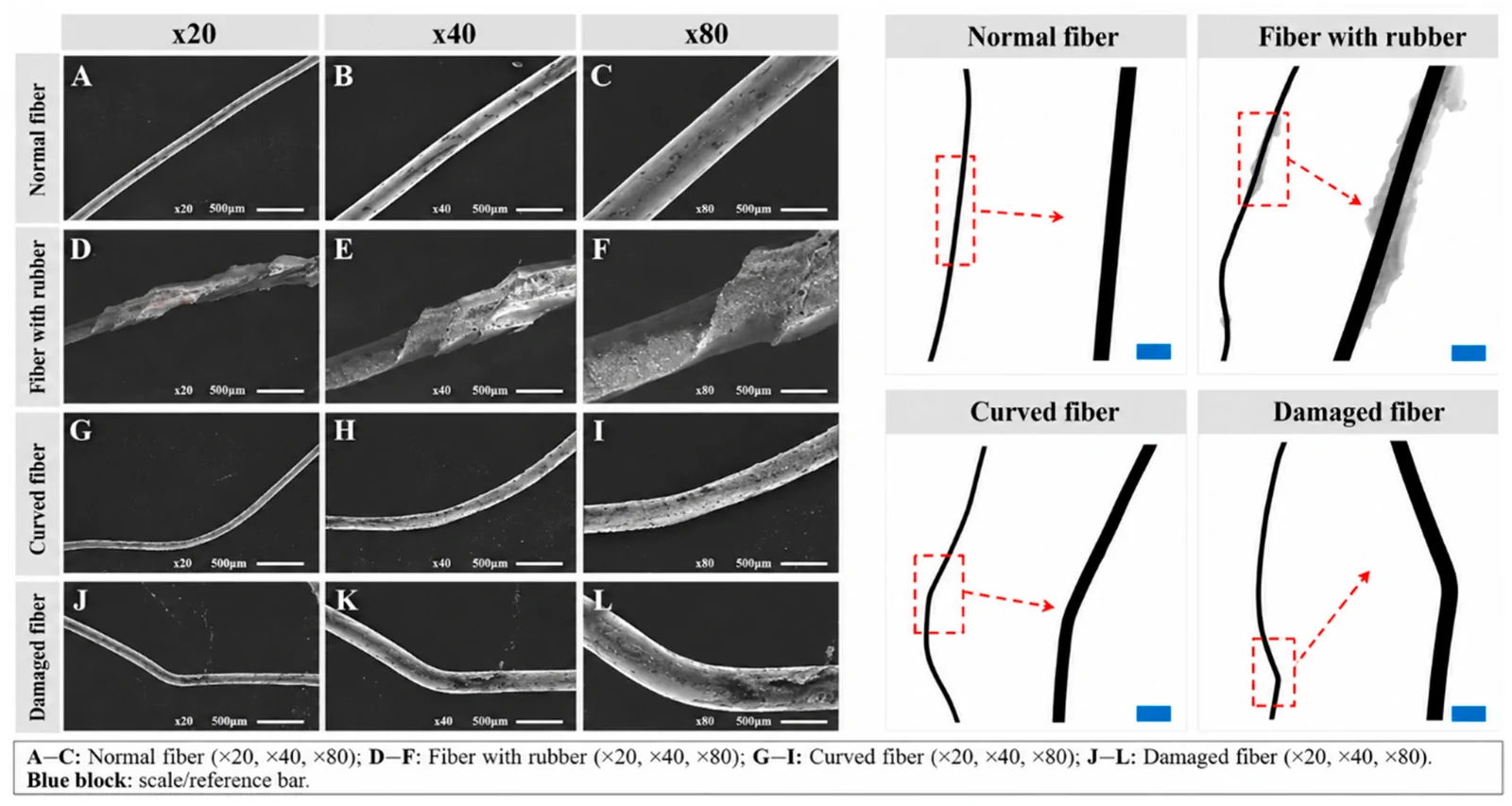
Original RSF surface characterization: SEM micrographs (**left**) and micro-CT morphology (**right**). Scale bars and labels remain those of the source images.

Figure 1 documents the heterogeneous RSF morphology, including smooth, curved, twisted, locally damaged, and rubber-contaminated surfaces. This variability can act through competing mechanisms: geometric irregularity may enhance mechanical anchorage, whereas limited embedment length, surface contamination, and fiber entanglement may reduce effective crack bridging. Michalik et al. [18] showed that purification and refinement can yield recycled-fiber concrete with properties exceeding those of a manufactured-fiber counterpart. Together with the selected-fiber results of Caggiano et al. [10], this confirms that the morphology shown in Figure 1 characterizes the investigated supply rather than an intrinsic limitation of recycled steel fibers. Because tensile-property and density values differ among the source records, they are not used here to infer single-fiber forces or convert mass dosage into an asserted design volume fraction.

### 3.2. Mixture Proportions and Fresh-State Behavior

Table 2 summarizes the thesis mixture proportions. Each reinforced mixture contained 90 kg/m^3^ of total steel fibers, with RSF replacement levels of 0%, 50%, 67%, and 100% by fiber mass. Preserving workable self-compacting mixtures required adjustments to both the matrix and granular skeleton. Relative to I90R0, mixtures containing RSF generally used greater powder and water contents and a modified aggregate balance. The resulting comparisons therefore represent complete composite systems; fiber origin is not the only experimental variable.

**Figure 2:**
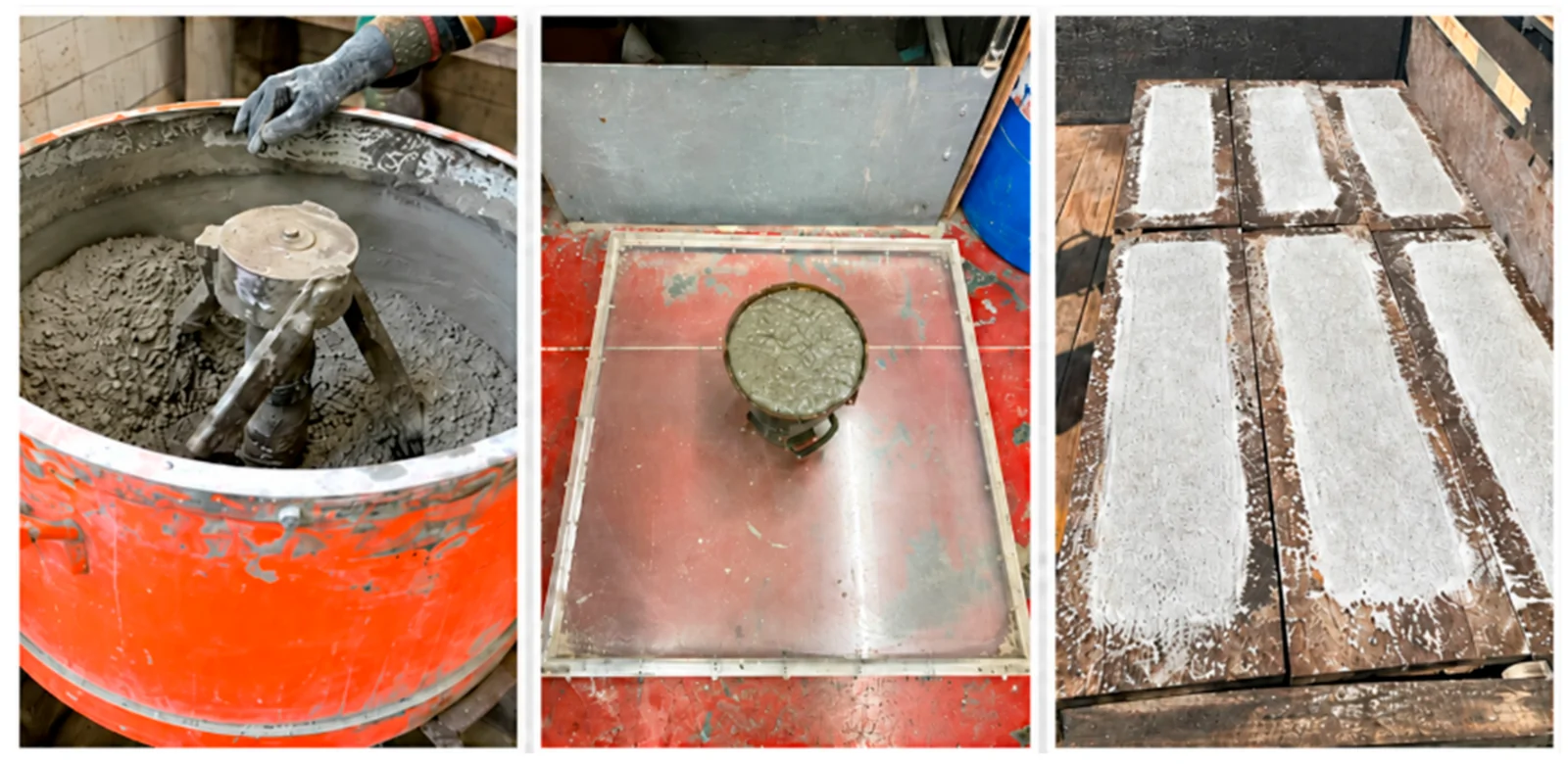
Original production photographs: mixing (**left**), slump-flow assessment (**center**), and cast beam specimens (**right**).

Aggregates were introduced from coarse to fine and mixed for approximately 1 min, after which water was added and mixing continued. Cementitious powders and limestone filler were then incorporated, and the fibers were introduced progressively by hand. Mixing continued for approximately 5 min after fiber addition until visual homogeneity was achieved [1]. Figure 2 documents the laboratory mixing sequence, slump-flow measurement, and specimen casting. This procedure provides a laboratory baseline; mixer capacity, batch volume, fiber feed rate, mixing energy, and sequence must be requalified for manufacturing-scale production.

The 2022 program reported a slump flow of approximately 600 mm, while the hybrid concrete used in the bond study had a reported T50 slump-flow time of 3.5 s [2,4]. These measurements characterize flowability under the corresponding laboratory conditions, not guaranteed passing ability through every reinforcement configuration. Slump flow is addressed in ASTM C1611/C1611M [19], while static segregation can be assessed separately by a column procedure such as ASTM C1610/C1610M [20]. Neither measurement substitutes for direct characterization of fiber distribution. For a thin dock shell, the qualified fresh-state window should therefore account for mold geometry, reinforcement congestion, flow path, temperature, placement time, bundling, and segregation. An additional 120 kg/m^3^ RSF mixture mentioned in the thesis is excluded because the complete screening dataset is unavailable.

Acceptable slump flow can coexist with unfavorable fiber orientation, particularly near corners, changes in section, and congested reinforcement. The reductions in filling and passing ability reported for steel-fiber SCC [13] therefore reinforce the need to couple fresh-state acceptance criteria with direction-sensitive, hardened testing of representative trial castings. Curing, demolding, cutting, and handling ages should likewise be linked to the specimens and components being qualified; the reported 28-day properties do not establish lifting or handling resistance at earlier ages.

## 4. Mechanical Performance and Fiber-Field Characterization

### 4.1. Elastic Modulus and Compressive Strength

**Figure 3:**
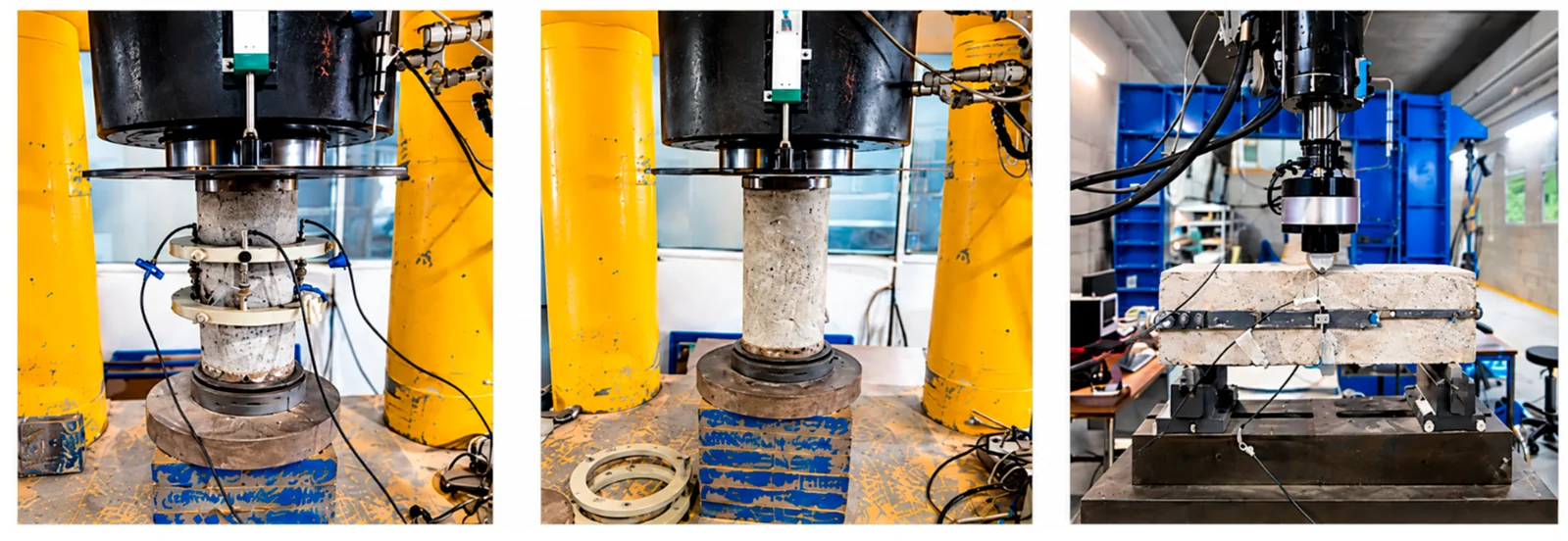
Original test photographs: elastic-modulus instrumentation (**left**), cylinder compression (**center**), and notched-beam flexure (**right**).

Twelve 150 × 300 mm cylinders, three per fiber system, characterized the elastic modulus. Ground ends reduced seating irregularities. The thesis reports a 1000 kN machine, loading at approximately 0.28 MPa/s, and three displacement measurements between rings over a 100 mm gauge length [1]. Equation (1) expresses chord modulus following the approach of ASTM C469/C469M [21]: S1 denotes stress at 50 microstrain, S2 denotes stress at 40% of maximum compressive stress, and ε2 denotes the strain at S2. With stresses in MPa and strains dimensionless, Ec is obtained in MPa. Compressive strength was measured with three cylinders per mixture in a 2000 kN machine under displacement control at 5 µm/s. Figure 3 presents the modulus, compression, and flexural setups.

(1) $$E_{c}=\frac{S_{2}-S_{1}}{\varepsilon_{2}-0.000050}$$

The 28-day mean compressive strengths ranged from 60.03 to 66.10 MPa, while elastic moduli ranged from 30.00 to 31.41 GPa (Table 3). Relative to I90R0, full replacement with RSF reduced the reported mean compressive strength by approximately 9.2% and the modulus by 4.5%. These differences are modest compared with the reductions observed in several post-cracking indicators. Although the reported cylinder CoVs are low, three specimens per mixture are insufficient to characterize production variability or establish a characteristic strength class. Differences in matrix composition also preclude attributing the observed changes solely to fiber type.

Compressive strength is sensitive to specimen fabrication, curing, end preparation, and test conditions, as emphasized by ASTM C39/C39M [22]. The relatively narrow strength and modulus ranges therefore support matrix-level comparison, but they do not replace direct qualification of fiber-controlled post-cracking behavior. This distinction is consistent with Aiello et al. [9], who observed limited sensitivity of compressive response to recycled-fiber reinforcement even when flexural performance changed appreciably.

### 4.2. Notched-Beam Flexure and Work Absorption

The flexural specimens measured 150 × 150 × 600 mm, with a 500 mm support span and a 25 mm midspan notch leaving a 125 mm ligament [1,2]. Three beams per mixture were tested at 28 days. Displacement control was 1 µm/s up to 0.1 mm midspan deflection and 3 µm/s thereafter. Both crack-mouth opening displacement (CMOD) and midspan deflection were recorded. Equation (2) gives the nominal residual flexural stress from center-point force Fj, beam width b, support span L, and remaining ligament depth $h_{\mathrm{sp}}$. The RILEM bending recommendation provides the methodological context [23]; the load and crack-opening histories are source measurements [1,2].

(2) $$f_{R,j}=\frac{3F_{j}L}{2b{h_{\mathrm{sp}}}^{2}}$$

**Figure 4:**
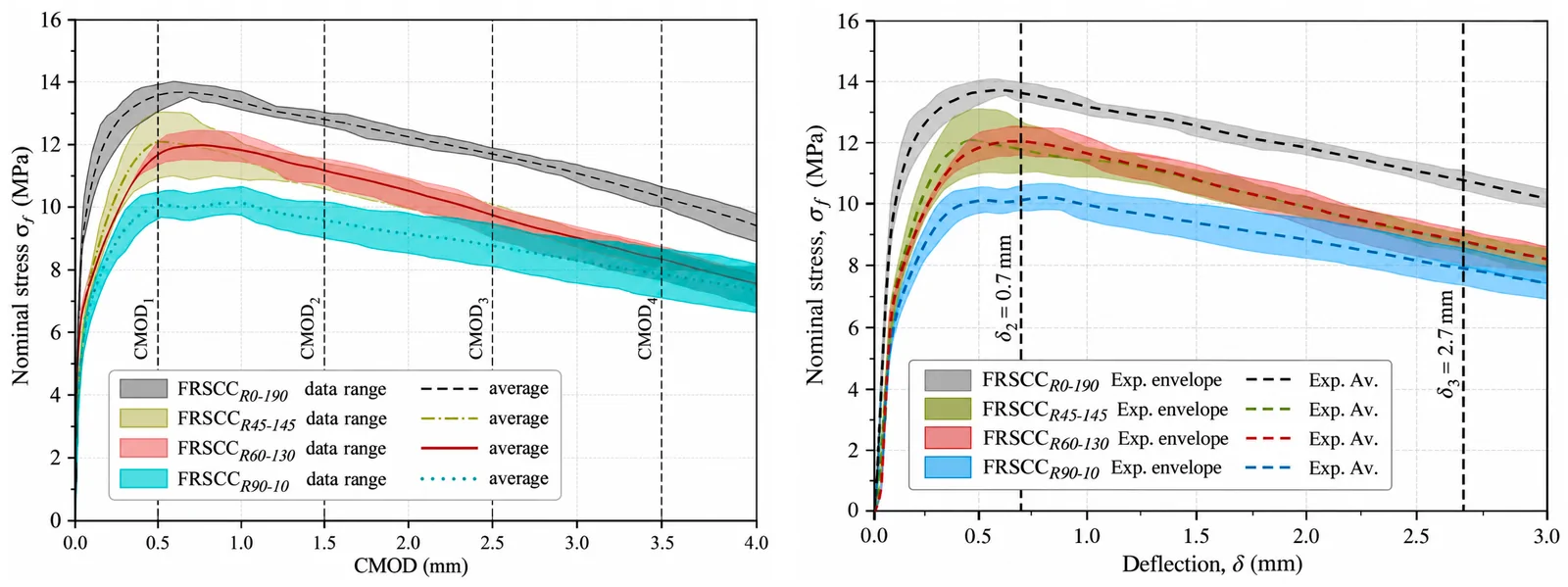
Nominal flexural stress responses against CMOD (**left**) and midspan deflection (**right**). Shaded envelopes represent experimental ranges, not newly estimated confidence intervals.

For the tested geometry, a center-point load of 1 kN corresponds to a nominal flexural stress of 0.32 MPa. Residual responses at CMOD values of 0.5, 1.5, 2.5, and 3.5 mm are distinguished from the additional 4.5 mm position discussed in the thesis. Figure 4 retains the original response curves and experimental envelopes. Initial cracking occurred at approximately 5 MPa, after which I90R0 developed the highest nominal resistance and sustained the strongest post-cracking response. The results demonstrate that similar matrix strengths do not imply an equivalent fiber-bridging capacity.

The reported load–deflection work integrated to 2.70 mm was 92.762, 77.967, 77.637, and 68.276 J for I90R0, I45R45, I30R60, and I0R90, respectively [2]. Because 1000 N·mm = 1 J, the hybrid mixtures retained approximately 84% of the reference work, whereas I0R90 retained approximately 74%. The hybrid means differ by only 0.330 J, equivalent to 0.36% of the I90R0 value. In the absence of uncertainty estimates for the integrated work, this difference is too small to support a robust ranking of the two hybrids.

Equation (3) defines the work U up to limiting midspan deflection $\delta_{\lim}$ by integration of applied force F (N) over displacement δ (mm), with a conversion factor of 10^−3^ from N·mm to J. The present limit is $\delta_{\lim}$ = 2.70 mm. This quantity is specimen-level flexural work, not the collision-energy capacity of a slab. ASTM C1609/C1609M [24] uses a different loading configuration and likewise does not make such work interchangeable with structural impact energy. That standard’s residual strengths, direct-tension values, and this notched-beam nominal response must be distinguished.

(3) $$U_{\lim}=10^{-3}\int_{0}^{\delta_{\lim}}F(\delta )d\delta$$

The lower beam work measured for I0R90 is consistent with reduced effective anchorage for the investigated recycled-fiber supply, but it should not be generalized to RSFRC as a material class. Hu et al. [11] reported comparable performance for selected manufactured–recycled hybrids, while longer recycled tire steel cords mobilized substantial resistance at larger crack openings [12]. Other hybrid fiber systems have also shown beneficial interactions under application-specific conditions [25]. These results support performance-based specification of residual response and fiber quality rather than a universal RSF replacement ratio.

### 4.3. Modified Splitting-Tension Response: Strength, Crack Opening, and Direction

**Figure 5:**
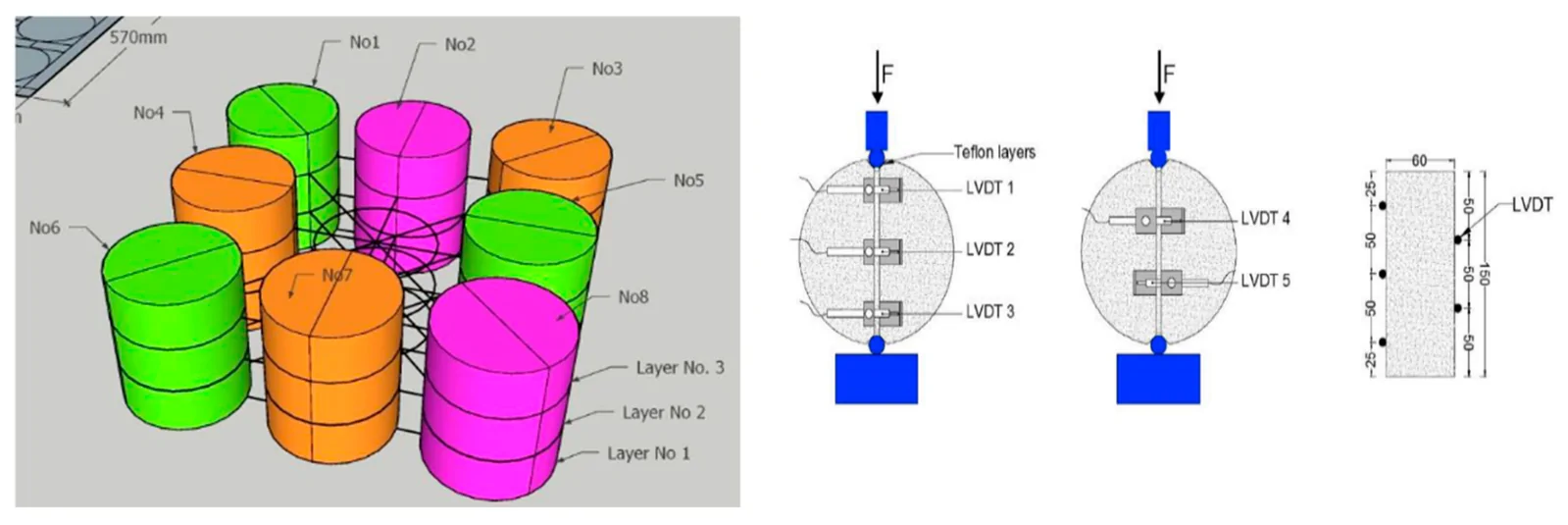
Splitting test documentation: core positions and depth sampling (**left**), and loading/LVDT arrangement (**right**).

The modified-splitting program sampled centrally cast nominal 570 × 570 × 240 mm panels using approximately 150 mm diameter cores, sectioned into nominal 60 mm disks at bottom, middle, and top depth positions [1,2,3]. The extraction description in [3] specifies eight cores per panel, three disks per core, and notches designed to localize the fracture plane. For the published nominal stress, the source reports a 120 mm effective diameter and 50 mm effective thickness across the notched section; these must not be confused with the unnotched disk dimensions. Nevertheless, ref. [3] gives incompatible overall disk/test counts (60, 96, and a statement of nine specimens per direction and mixture), so the exact statistical sample size behind each published aggregate response cannot be resolved from the available reporting. Figure 5 retains the documented extraction and instrumentation schemes. The current study does not recompute stresses, pool orientations, or claim a characteristic 1 or 3 mm resistance from these aggregate data.

Five LVDTs—three on one face and two on the opposite face—measured transverse crack opening, and the reported opening was obtained from their average. The test used a 1500 kN machine with displacement rates of 1 µm/s up to 2 mm, 2 µm/s from 2 to 3 mm, and 4 µm/s thereafter [3]. Notches were oriented parallel (θ = 0°) or perpendicular (θ = 90°) to the concrete flow direction. These angles describe notch orientation and are distinct from the fiber-axis angles used in the CT analysis.

**Figure 6:**
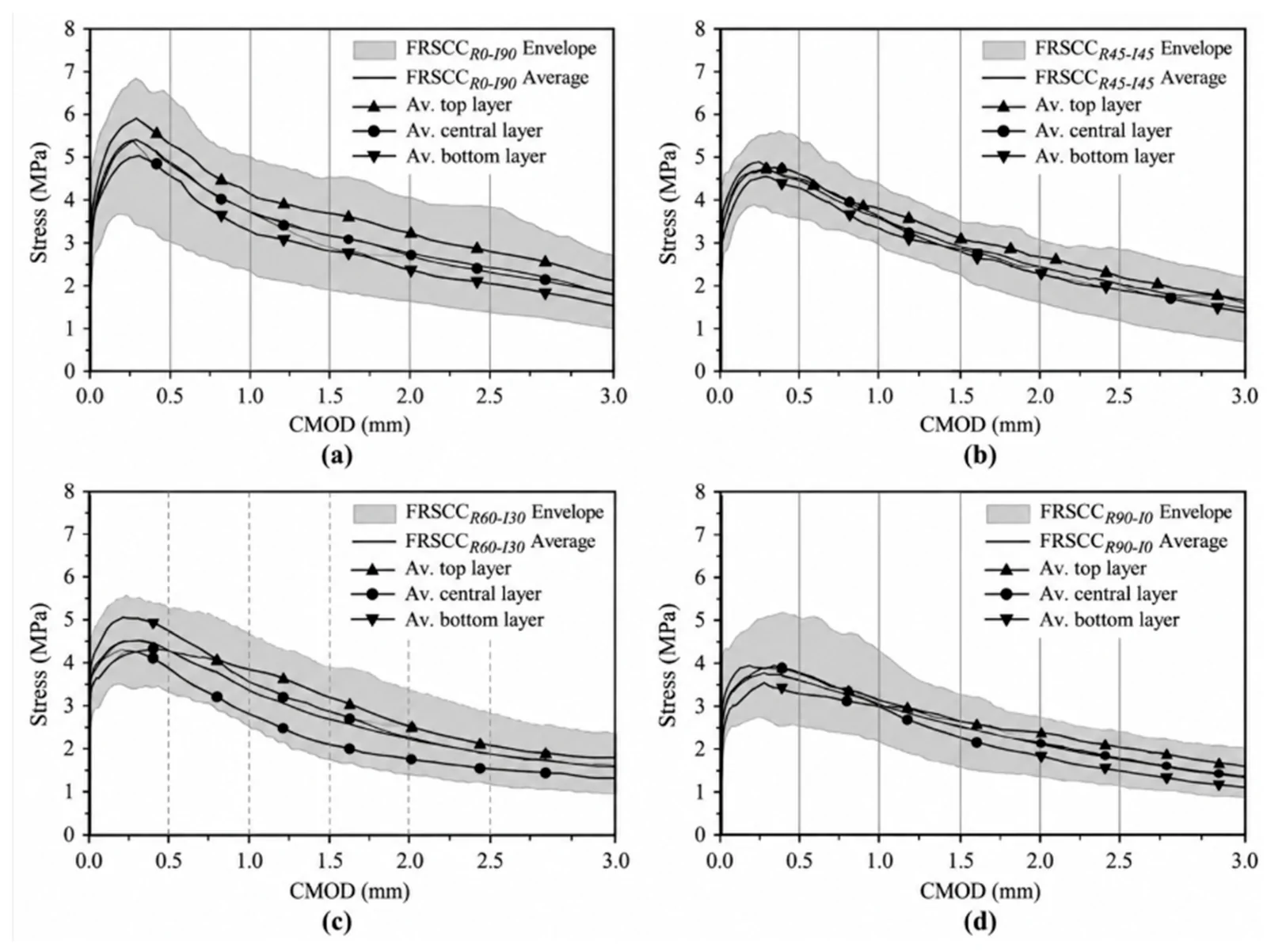
Original splitting nominal stress–crack-opening responses, including depth comparisons and experimental envelopes: (**a**) ${FRSCC}_{R0-I90}$; (**b**) ${FRSCC}_{R45-I45}$; (**c**) ${FRSCC}_{R60-I30}$; (**d**) ${FRSCC}_{R90-I0}$.

The notched planes were defined relative to radial concrete flow: θ = 0° denotes a crack plane parallel to flow and θ = 90° a perpendicular crack plane. The originally reported response depends on both orientation and depth [3]; consequently, the aggregate means at 1 and 3 mm in Table 4 must not be interpreted as orientation-independent design values. The source provides coefficients of variation for those means (14–27% at 1 mm and 25–30% at 3 mm) but does not establish a consistent independent sample count for each screened response. No standard errors, confidence intervals, pooled standard deviations, or statistical pass probabilities are reconstructed. The derived selection thresholds therefore describe only the reported nominal benchmark, and the least favorable relevant casting direction requires direct verification. Figure 6 compares the original splitting nominal stress–crack-opening responses of the industrial, hybrid, and recycled systems, including the depth-dependent averages and experimental envelopes.

After an approximately linear precracking response, crack initiation produced a small load drop followed by fiber-mediated resistance. Table 4 reports the published peak, residual, and integrated stress–opening indicators together with their source CoVs. Increasing RSF replacement reduced the maximum nominal splitting stress from 5.63 MPa for I90R0 to 3.73 MPa for I0R90. At a 1 mm crack opening, however, the corresponding values were 3.94 and 3.06 MPa, a reduction of approximately 22% compared with approximately 34% at peak stress. The replacement effect therefore changes the shape of the post-cracking response rather than simply scaling the entire curve by a constant factor.

At a 1 mm opening, I45R45 and I30R60 retained 3.77 and 3.50 MPa, respectively. Their integrated nominal stress–opening measures up to that opening were 4.26 and 4.10 N/mm, compared with 4.74 N/mm for I90R0 and 3.40 N/mm for I0R90 [2]. Equation (4) defines the nominal splitting stress–opening integral G up to a selected crack width wlim, using nominal splitting stress $\sigma_{N}$ (MPa) and opening w (mm). The result has units N/mm. This modified-splitting indicator is geometry-specific, is not the conventional cylinder splitting tensile strength, and must not be equated with flexural work in J.

(4) $$G_{\lim}=\int_{0}^{w_{\lim}}\sigma_{N}(w)dw$$

All mixtures exhibited declining resistance at larger crack openings. Consequently, ranking by peak stress, residual stress at 1 mm, residual stress at 3 mm, or an integrated stress–opening quantity can lead to different conclusions regarding the degree of performance retained as RSF content increases.

Directional testing showed greater post-cracking resistance when the notch was parallel rather than perpendicular to the casting flow [1,3]. The most plausible interpretation is that the casting-induced fiber field created a more favorable population of fibers intersecting one crack plane. Under this casting arrangement, depth-to-depth differences were generally smaller than orientation-related differences. Barnett et al. [26] likewise reported casting-dependent flexural response in round UHPFRC panels, where fiber alignment favorable to radial-crack bridging increased strength. Although their matrix and geometry differ from those considered here, the governing principle is transferable: orientation is beneficial only relative to the crack plane that must be bridged. Component qualification should therefore preserve casting coordinates and test the directions most likely to govern cracking.

### 4.4. Digital Image Analysis: Effective Fiber Intersections and Spatial Variability

The DIA procedure examined prepared cut or fracture surfaces and separated steel-fiber intersections from the concrete background using image processing followed by manual verification [1,3] with ImageJ software (version 1.54p, National Institutes of Health, Bethesda, MD, USA).

**Figure 7:**
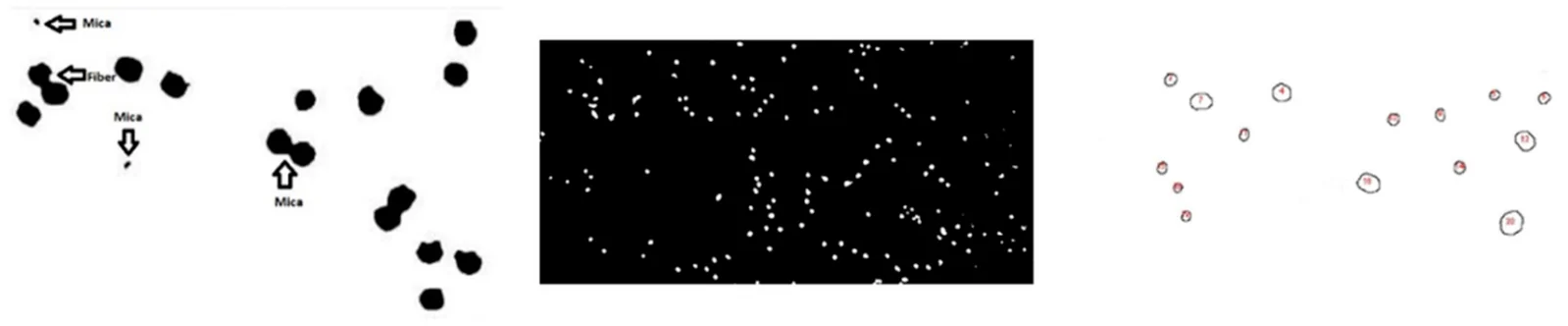
Original DIA evidence: identification of potentially confusing particles (**left**), segmented fiber intersections (**center**), and manually identified intersections (**right**).

Figure 7 shows the original segmentation and identified intersections. Bright mineral particles can be misclassified as steel, while curved fibers can generate ambiguous two-dimensional intersections. Consequently, the measured count depends on surface preparation, thresholding, and the operational definition of an effective fiber. DIA is therefore a diagnostic descriptor of the local fiber field rather than a direct measure of tensile resistance.

Mean effective intersection counts reported in 2022 were 174 (CoV 19%) for I90R0, 245 (9%) for I45R45, 241 (17%) for I30R60, and 250 (9%) for I0R90 [2]. The RSF-containing systems therefore exhibited more counted intersections despite lower peak nominal splitting resistance. Fiber count alone cannot quantify the crack-bridging capacity when fiber length, anchorage, inclination, curvature, and surface condition differ. The smoother descending response observed in some RSF-containing specimens is compatible with more distributed bridging, but it does not imply equal force transfer by each counted fiber.

The thesis also provides individual records for hybrid systems. Across the complete reported records, disk-level effective-count ranges were 151–286 for I45R45 at θ = 0° and 122–245 for I30R60 at θ = 90° [1]. Because disks are nested within cores and panels, they cannot be treated as independent production batches. The observed ranges nevertheless demonstrate that acceptable average fiber content does not eliminate local variability. Production qualification should therefore relate local fiber-field descriptors to local residual mechanical response instead of adopting an unsupported universal minimum intersection count.

### 4.5. Micro-CT Characterization and Limits of Orientation Transfer

**Figure 8:**
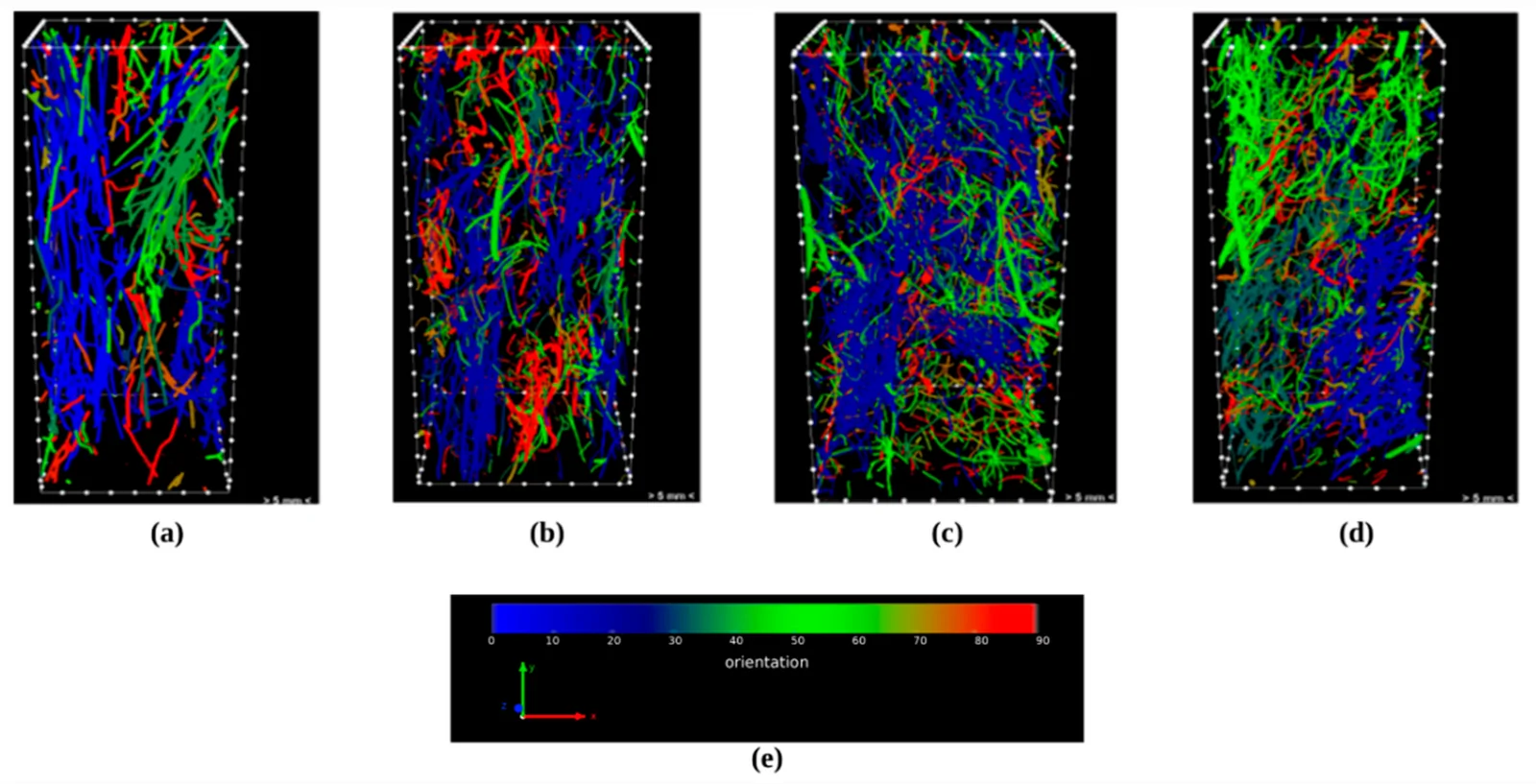
Colour-coded spatial distributions of steel fibers according to their orientation: (**a**) FRSCC_R0-I90 (industrial steel fibers); (**b**) FRSCC_R45-I45 (hybrid steel fibers); (**c**) FRSCC_R60-I30 (hybrid steel fibers); (**d**) FRSCC_R90-I0 (recycled steel fibers); and (**e**) fiber orientation colour scale (0–90°) and reference coordinate axes.

The CT program analyzed one representative specimen from each mixture, with approximate dimensions of 100 × 45 × 40 mm [3]. The imaging workflow used a VivaCT 80 system at 26 µm resolution and higher-resolution SkyScan 1272 imaging at 5 µm. Three-dimensional reconstruction enabled direct visualization of fiber trajectories and spatial organization without reducing each fiber to a single planar intersection. Figure 8 retains the reconstructed volumes and original orientation convention.

The I90R0 volume exhibited a more organized directional pattern, whereas increasing RSF content produced a broader orientation distribution and more visible local clustering. CT therefore captures information that DIA cannot fully recover, particularly for bent fibers and clustered groups whose two-dimensional intersections may appear acceptable. With only four scanned volumes, the results support qualitative comparison but do not separate the individual effects of inclination, embedment length, and clustering. The full-scale wall observations of hosseinmostofi et al. [1,3] further emphasize the need for production-scale sampling; a small CT volume cannot certify fiber orientation throughout a complete dock shell.

A strong mixture-level association between DIA and CT effective-fiber estimates was reported (R^2^ = 0.964) [3]. However, the correlation is based on only four mixture-level points and on different definitions of fiber effectiveness and therefore does not constitute an independent calibration for production control. The published CT orientation bins also sum to 100%, 99.0%, 96.9%, and 89.8% for the four systems. No renormalization or orientation reduction factor is introduced in the present analysis. The defensible use of the CT evidence is comparative and diagnostic: it identifies casting-sensitive fiber fields and supports representative residual-strength testing in the mechanically relevant directions.

## 5. Marine Conditioning and FRP–Concrete Bond Response

### 5.1. Specimens, Conditioning, and Loading

**Figure 9:**
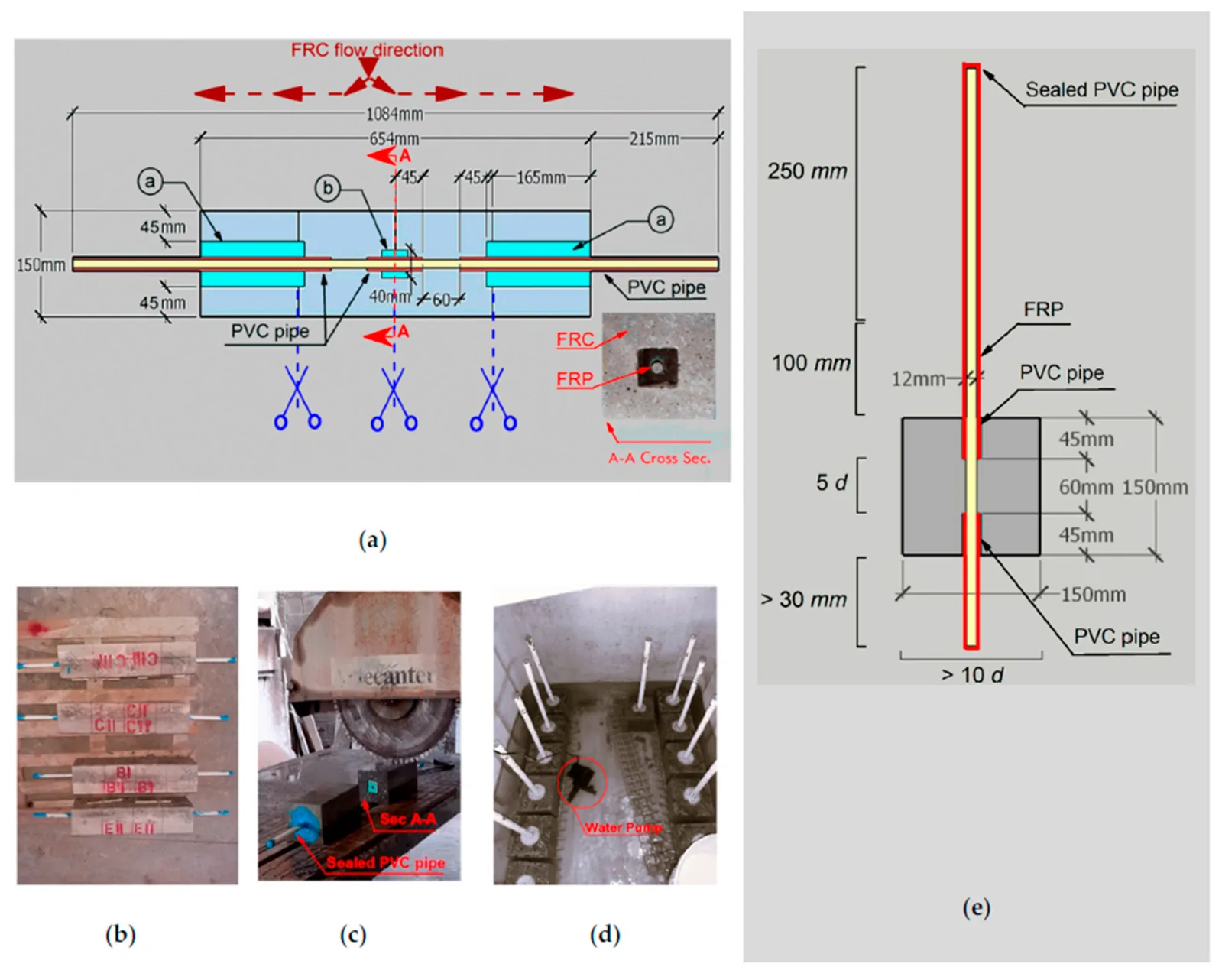
Pull-out specimen preparation and experimental details: (**a**) schematic of the casting mold, FRC flow direction, and specimen extraction locations; (**b**) prepared and labeled concrete specimens; (**c**) specimen cutting procedure and sealed PVC pipe arrangement; (**d**) water-conditioning setup for the specimens; (**e**) schematic of the pull-out specimen geometry, FRP bar configuration, PVC pipes, and bonded-region dimensions.

The bond program evaluated I45R45 concrete with two distinct 12 mm FRP products: a sand-coated, helically wrapped BFRP bar and a ribbed GFRP bar [4]. Twenty-four 150 mm cubes formed eight groups of three, combining bar type, conditioning, and loading protocol. Centered bars had a 69 mm nominal clear cover, giving a cover-to-bar-diameter ratio of 5.75. Plastic sleeves isolated the prescribed bonded length. Figure 9 preserves the source specimen drawings and photographs, including the 5d bonded region.

Specimens were cast in a 654 × 150 × 150 mm mold with the bar aligned with the casting direction, demolded after 48 h, and cut after 7 days. Unbonded bar regions were protected with PVC and epoxy during conditioning. Half of the specimens were tested at 28 days; the remainder were exposed for 24 months to simulated seawater at 23 °C after initial curing. The solution contained 24.53 g NaCl, 5.20 g MgCl_2_, 4.09 g Na_2_SO_4_, and 1.16 g CaCl_2_ per liter of deionized water [4]. ASTM D1141 [27] provides a reference composition for substitute ocean water; laboratory conditioning does not reproduce biological activity, wetting–drying cycles, mechanical damage, or the complete exposure history of a marine structure. During conditioning, containers were covered, the solution level was maintained, and circulation was provided every 3 h.

Static pull-out testing was performed in a 200 kN machine at 0.021 mm/s with data acquisition at 2 Hz. A ball joint minimized unintended bending. Three LVDTs around the loaded end measured relative displacement, and an additional LVDT measured free-end slip. Cyclic specimens were subjected to a target of 10^6^ cycles between 15% and 30% of the relevant peak bond load and were subsequently loaded monotonically to pull-out failure [4]. The post-cycle curves therefore describe the residual bond after one defined cyclic history; they do not establish a fatigue endurance limit for other stress ranges or environmental conditions.

The average apparent bond stress in Equation (5) is calculated from the applied force P, bar diameter d, and bonded length, with P in N and geometric quantities in mm, yielding τ in MPa. The value represents an average over the cylindrical interface rather than the local bond-stress distribution. Equation (6) defines the conditioned-to-control mean peak-stress ratio (%) for a given bar and loading protocol. Because the 24-month conditioned specimens and 28-day controls are not age matched, this ratio is a comparative experimental indicator and must not be interpreted as an isolated seawater-degradation factor.

(5) $$\tau =\frac{P}{\pi dl_{b}}$$

(6) $$R_{\tau }=\frac{\tau_{\mathrm{cond}}}{\tau_{\mathrm{ctrl}}}\times 100\%$$

ASTM D7913/D7913M [28] explicitly treats pull-out testing as a comparative characterization method and cautions against direct use of the measured bond stress to establish development length. Surface morphology is central to this response: Katz [29] demonstrated that FRP surface treatment affects both peak bond and post-peak behavior. Accordingly, the present BFRP–GFRP comparison reflects the complete bar–surface–concrete interface, including coating and rib geometry, rather than fiber type alone.

### 5.2. Static and Post-Cycle Bond Response

**Figure 10:**
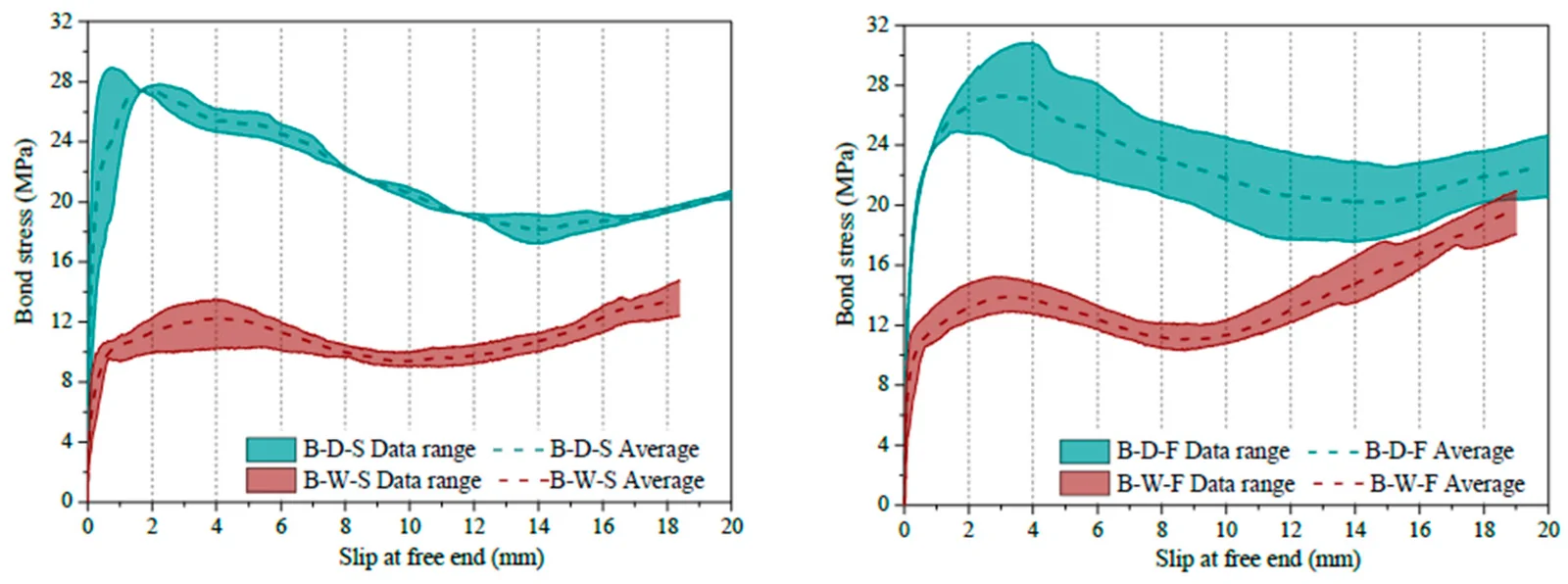
Original BFRP free-end bond–slip curves for unconditioned and seawater-conditioned specimens: static loading (**left**) and static loading after cycling (**right**).

Table 5 summarizes peak bond stress, slip at peak, bond stress at 0.05 mm free-end slip, conditioned-to-control peak-stress ratios, and specimen-level failure modes. Figure 10 shows the BFRP bond–slip curves for both loading histories. The 28-day control groups reached mean peak stresses of 27.39 and 27.30 MPa under static and post-cycle testing, respectively, whereas the 24-month conditioned groups reached 12.14 and 13.90 MPa. The corresponding conditioned/control mean ratios are 44% and 51%, with greater slip at peak in the conditioned groups. The contrast is substantial for the tested interface; however, because age and exposure changed simultaneously, the experimental design does not isolate the fraction attributable to seawater from age-dependent matrix/interface evolution.

**Figure 11:**
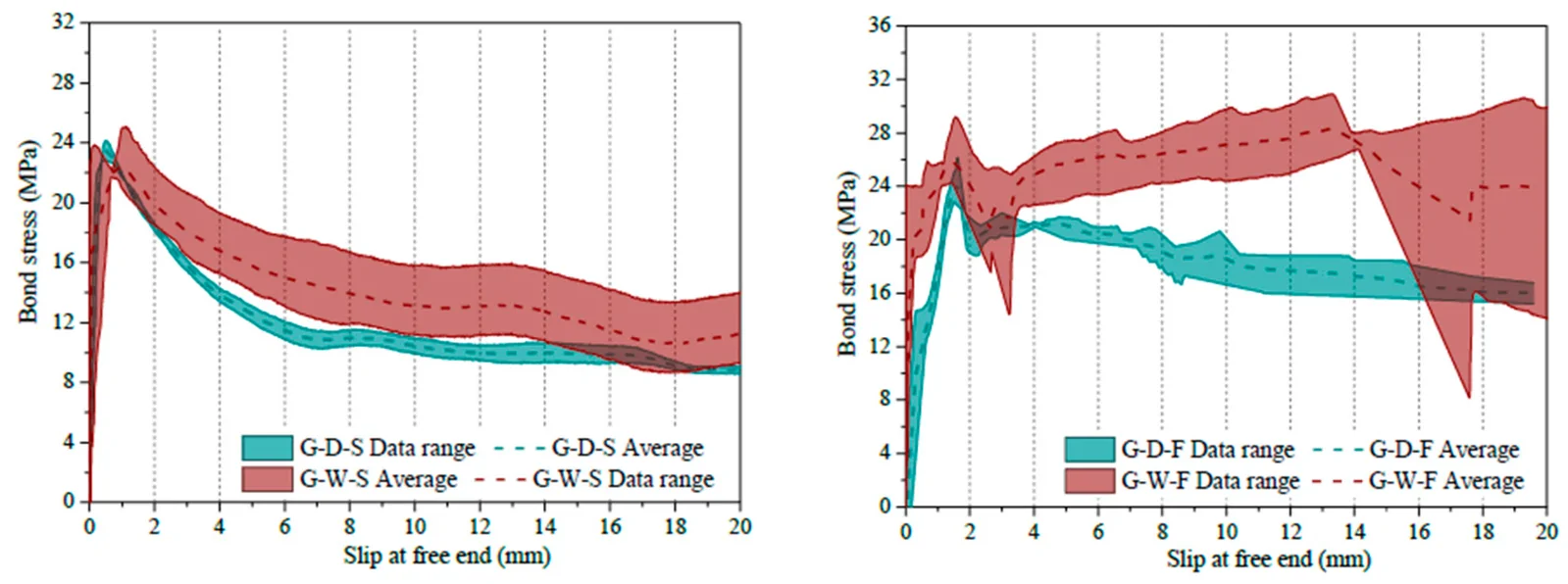
Original GFRP free-end bond–slip responses under static loading (**left**) and after cycling (**right**), comparing unconditioned and conditioned groups.

For the tested GFRP product, the mean static peak bond stress was 23.50 MPa in the 28-day control group and 22.46 MPa in the 24-month conditioned group, while the post-cycle means were 24.16 and 25.78 MPa. The corresponding conditioned/control ratios are approximately 96% and 107% (Figure 11). The value above 100% is not evidence that seawater improves the GFRP bond. The groups are not age matched, so matrix maturation, specimen variability, exposure effects, and interface evolution cannot be separated by this experimental design.

Small-slip response provides information that is not captured by peak stress. For BFRP, the stress at 0.05 mm free-end slip decreased from 8.61 to 4.00 MPa under static loading and from 6.50 to 4.60 MPa after cycling. For GFRP, the corresponding control/conditioned values were 6.50/12.74 MPa and 1.40/11.15 MPa [4]. These values characterize early force transfer in the tested interfaces. Although the source refers to them as design-bond stresses, they are treated here strictly as measurements at a specified slip; no code-level anchorage resistance is assigned. In particular, the large conditioned/control difference in the post-cycle GFRP group cannot be isolated as a seawater effect because age and exposure changed simultaneously.

Failure mode provides a second constraint on interpretation. All six conditioned BFRP specimens failed by bar slip, whereas all three 28-day post-cycle BFRP controls exhibited concrete splitting. Each GFRP post-cycle group contained two splitting failures and one slip failure [4]. Similarity or contrast in peak stress therefore does not demonstrate preservation of the same failure mechanism or adequate confinement in a thin shell. Wang and Belarbi [30] likewise reported that cycling can alter both strength and brittleness in an FRP/fiber-reinforced-concrete interface. Qualification should therefore consider small-slip response, peak stress, post-peak behavior, and failure mode collectively and should select the complete bar–surface–concrete system rather than FRP fiber type in isolation.

### 5.3. Surface Condition, Failure Mechanisms, and Transfer to Thin Shells

**Figure 12:**
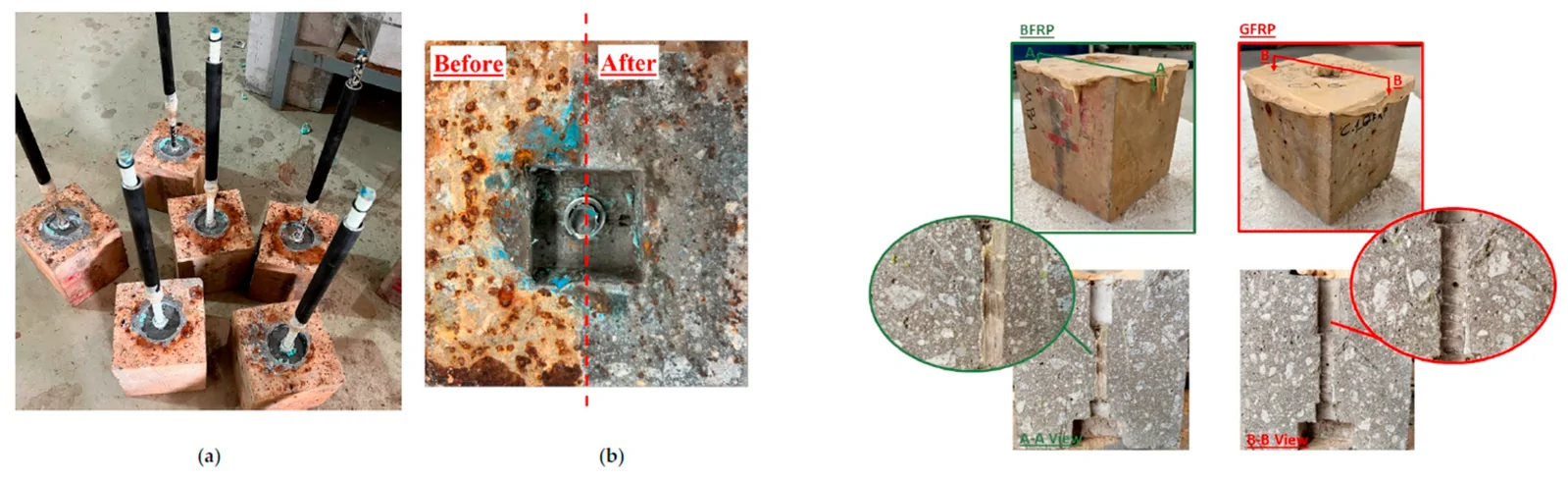
Original marine and failure observations: visible fiber corrosion before and after surface cleaning (**left**, (**a**,**b**)), and post-test concrete/bar-interface damage (**right**).

Figure 12 retains the original post-exposure and post-test observations. Visible rust on exposed steel fibers confirms that a composite containing steel fibers and FRP bars should not be described as corrosion-free; conversely, surface staining alone does not quantify retained residual tensile capacity. The observations distinguish between bar slip, interface damage, and concrete splitting. Changes in FRP surface condition together with altered failure mode are consistent with interface degradation, although concrete confinement remains a competing mechanism [4]. Fiber restraint of splitting is therefore part of the interface system. Leone et al. [31] reported favorable bond behavior in concrete containing recycled steel fibers, but with steel reinforcement and a different matrix, while Frazão et al. [32] observed slightly greater corrosion susceptibility of recycled fibers without a significant post-cracking penalty under their conditions. These findings reinforce the need to measure retained mechanical performance directly rather than infer it from visible corrosion alone.

A geometric comparison illustrates the transfer problem. A centered 12 mm bar in a 100 mm shell permits at most a 44 mm nominal clear cover or a cover-to-diameter ratio of 3.67 before placement tolerance, whereas the 150 mm test cube provided a ratio of 5.75. Thus, the cube results do not establish equivalent anchorage or splitting confinement in a thin shell. A local rib, revised reinforcement arrangement, or detail-specific anchorage test may be necessary. The thesis and bond study also report analytical long-term ratio projections, which are extrapolations rather than measured 50-year resistance.

The bond evidence therefore establishes a product-specific qualification priority rather than a generic ranking of FRP materials. For the tested I45R45 concrete, the GFRP interface warrants further detail-specific investigation, whereas direct transfer of the tested BFRP interface would require stronger justification. The present results do not rank all commercial BFRP and GFRP products and do not qualify prestressing anchorages, sustained-stress durability, cracked concrete, or cyclic wetting–drying exposure.

## 6. Structural Slab Response Under Drop-Weight Impact

### 6.1. Slab Manufacture and Instrumented Impact Test

**Figure 13:**
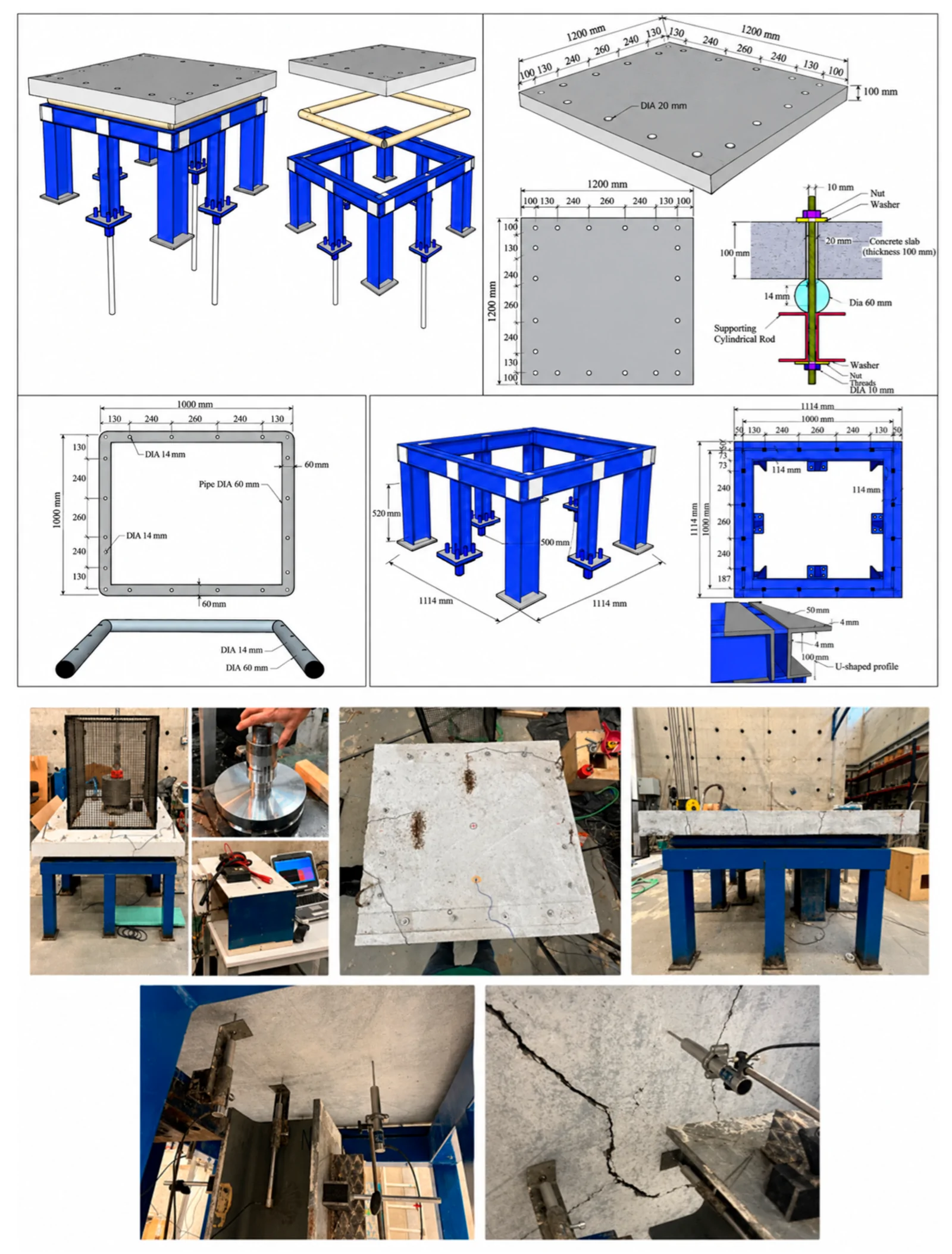
Original instrumented slab-impact apparatus, support arrangement and measurement details. The drawing represents the test rig, not a finite-element model.

The quantitative component-scale evidence analyzed in this article comprises four documented 4.0 m drop-weight tests on 1200 × 1200 × 100 mm slabs, one specimen of each of plain SCC, I90R0, I45R45, and I0R90, tested at 28 days [1,2,3]. The reinforced compositions contained 90 kg/m^3^ of steel fibers but differed in their matrix formulations. Before selecting the higher drop height, exploratory impacts were undertaken at approximately 2.5 m. No visually discernible damage was observed in those preliminary trials, prompting an increase to 4.0 m to investigate the slab response under a more demanding impact condition. The quantitative records and sufficiently documented damage measurements for the 2.5 m trials are not available for the present re-analysis; therefore, this observation is provided solely as the rationale for the selected loading level, not as a quantified lower-height result, a demonstrated no-damage threshold, or statistical replication. All impact comparisons and component-gate illustrations in this article use only the four documented 4.0 m tests. Figure 13 presents the support arrangement and purpose-built University of Minho apparatus, in which a 105 kg projectile was released electromagnetically. This component-scale test differs from standardized repeated-drop testing on small specimens.

Five LVDTs below each slab measured the vertical displacement history, with the reported mean used as the slab-deflection indicator; the 1000 kN projectile load cell measured contact force, and a ±10,000 g accelerometer 300 mm from the impact center measured local acceleration. The source states a sampling frequency of 100 kHz [1]. For projectile mass m = 105 kg, drop height h = 4.0 m, and gravitational acceleration g = 9.81 m/s^2^, Equation (7) gives the nominal incident potential energy of 4.12 kJ and an ideal impact speed of 8.86 m/s, neglecting dissipative effects during release. These calculated loading quantities are neither directly measured impact velocity nor measured absorbed energy.

The available records do not fully specify sensor calibration, analog anti-alias conditioning, inter-channel synchronization, filtering procedures and cutoff frequencies, baseline treatment, or peak-extraction intervals. Consequently, the force, acceleration, and displacement histories are presented as reported in the source record [1], without new digital signal processing, impulse integration, or absorbed-energy estimates. The force peaks of approximately 110–286 kN and acceleration magnitudes of approximately 297–780 g lie below the instruments’ nominal stated measurement limits; this is a basic range-consistency observation, not independent verification of waveform fidelity or a dynamic equilibrium check. In particular, a projectile-mounted load cell and an off-center slab accelerometer do not measure kinematics of the same body, so equating their separately recorded peak values through F = ma would be inappropriate. Verification of the high-frequency time histories requires raw synchronized channels, sensor calibration documentation, filtering/baseline metadata and the test-specific kinematics.

(7) $$E_{\mathrm{imp}}=mgh,\;v_{\mathrm{imp}}=\sqrt{2gh}$$

### 6.2. Damage Development and Deflection Response

**Figure 14:**
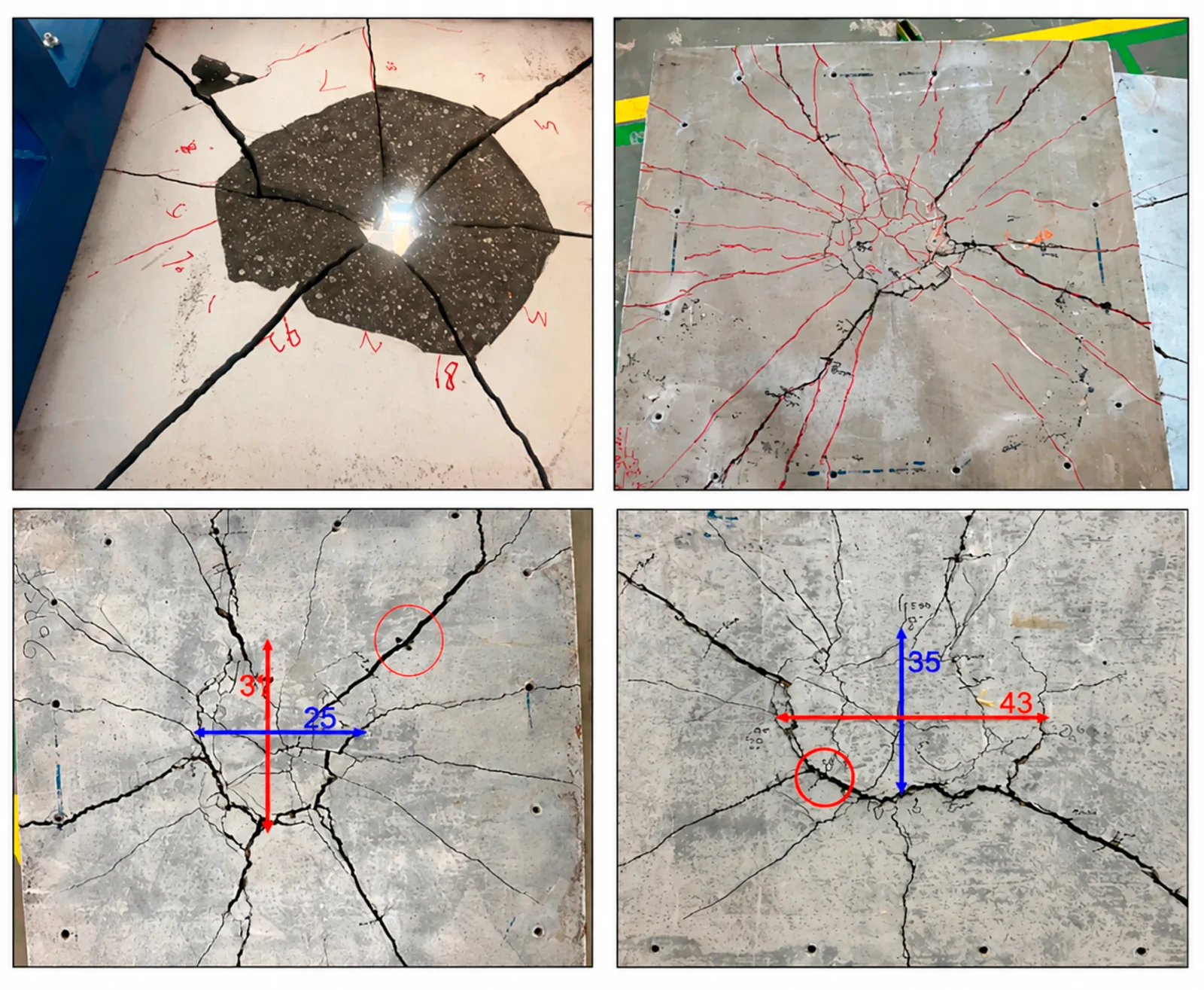
Post-impact bottom-face photographs: plain SCC (**upper left**), I90R0 (**upper right**), I45R45 (**lower left**) and I0R90 (**lower right**).

The plain SCC slab experienced perforation at the impact point, front-face spalling, and rear-face scabbing. In contrast, all three fiber-reinforced slabs developed distributed radial cracking without reported perforation or rear-face scabbing (Figure 14).

**Figure 15:**
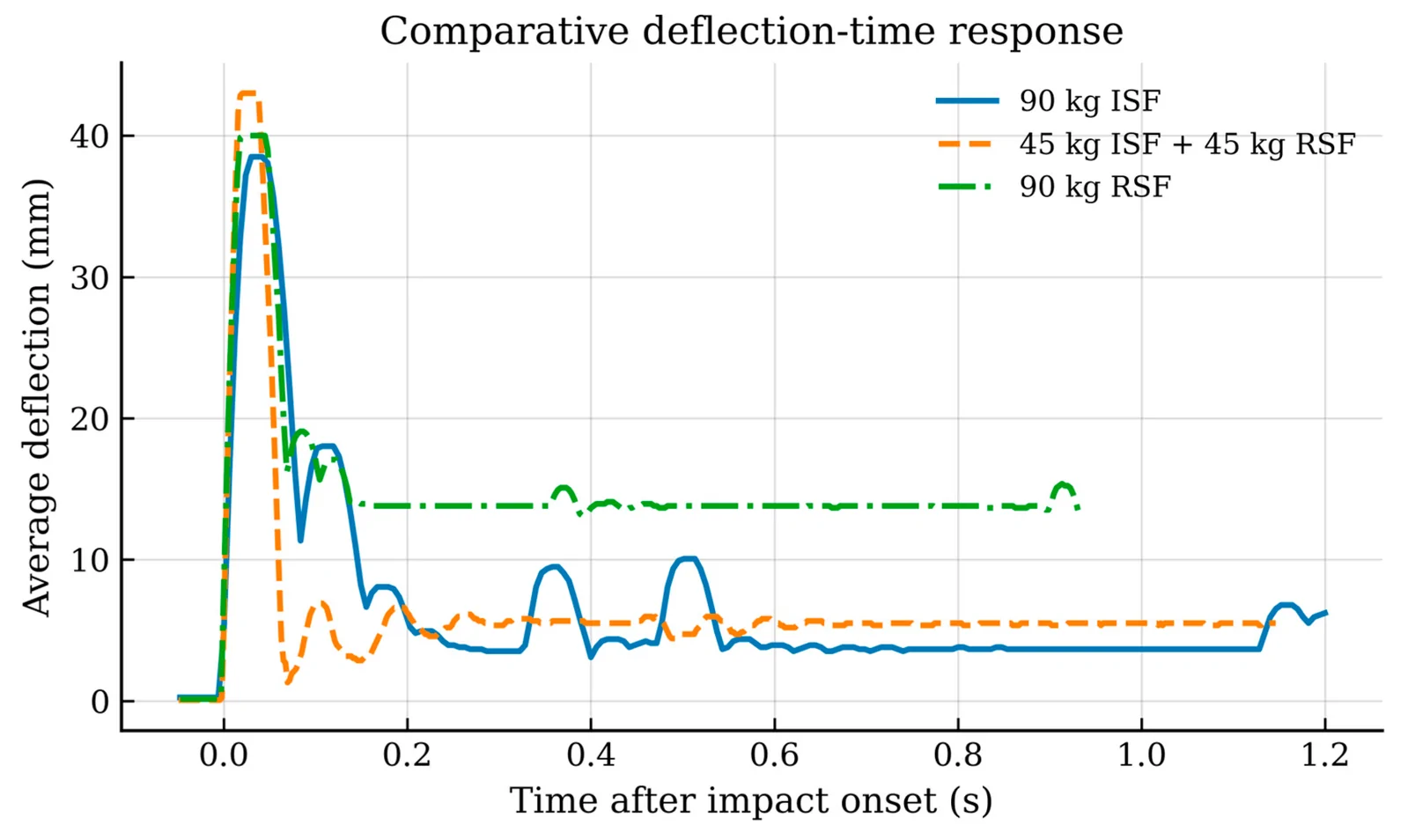
Original average bottom-LVDT deflection histories for the three fiber-reinforced slabs, aligned to impact onset.

Table 6 and Figure 15 distinguish peak from terminal average deflection. I90R0 reached approximately 38–39 mm and retained 3.5–4.0 mm after the event. I45R45 developed the largest peak deflection, approximately 42–43 mm, but retained only 5–6 mm, whereas I0R90 reached 39–40 mm and retained 13–14 mm. The recycled-only slab therefore exhibited a lower peak deflection than the hybrid but substantially greater permanent deformation. Based on the reported ranges, the terminal-to-peak deflection ratio was approximately 9–11% for I90R0, 12–14% for I45R45, and 33–36% for I0R90. These ratios summarize the reported ranges and are neither statistical intervals nor measures of energy recovery. The plain slab cannot be compared on the same basis because perforation disrupted the deflection response.

The reported damaged-zone area and diameter also produce different relative rankings for I45R45 and I90R0 (Table 6). Because the measurement boundaries are not documented sufficiently to reconstruct the damaged area from the marked photographs, a circular-area conversion is not introduced. Maximum recorded crack widths were 10 mm for I90R0 and 9 mm for both RSF-containing slabs [5]. The specimen with the smallest terminal deformation therefore did not exhibit the smallest individual maximum crack width. Crack localization and distributed deformation must be interpreted together. For a floating shell, the logical continuation of this test is post-impact leakage and residual-capacity assessment because fragment containment does not demonstrate preservation of the shell’s barrier function.

### 6.3. Contact Force, Acceleration, and Comparative Interpretation

**Figure 16:**
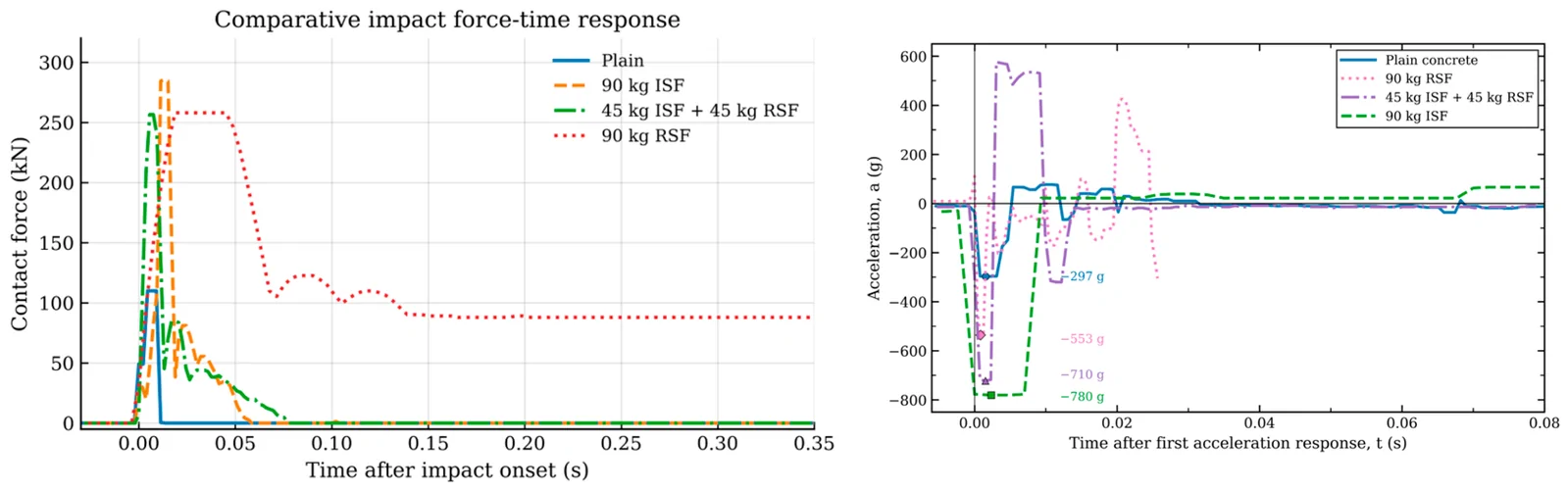
Original contact-force histories (**left**) and acceleration histories (**right**). Acceleration is local to the sensor 300 mm from impact; the source time origins and sign conventions are retained.

Peak contact forces were 110.00 kN for plain SCC and 285.83, 256.52, and 258.00 kN for I90R0, I45R45, and I0R90, respectively (Figure 16). The low peak recorded for plain SCC accompanied severe localized failure and therefore does not indicate superior impact performance. I90R0 transmitted the largest force while limiting terminal deformation. I0R90 showed a more sustained post-peak force history; without a complete motion and energy balance, however, this feature cannot be interpreted as greater absorbed energy.

The dominant negative acceleration magnitudes were 296.79 g for plain SCC, 780.26 g for I90R0, 710.25 g for I45R45, and 552.62 g for I0R90. Their ordering differs from that of peak deflection because the accelerometer records a local transient response rather than a single measure of global resistance. Neither low acceleration nor low contact force constitutes an acceptance criterion when accompanied by severe damage. Likewise, the 1.48 kN difference between the I45R45 and I0R90 peak forces is too small to support a meaningful ranking. Because the projectile force and off-center slab acceleration refer to different bodies and measurement locations, combining their separate peaks through F = ma would not provide a valid dynamic equilibrium check. Impulse, energy transfer, and dynamic stiffness require synchronized histories, appropriate kinematics, and boundary-reaction information.

The documented 4.0 m impact evidence comprises one observation per tested composition and excludes I30R60; it therefore cannot support a statistical impact ranking or a project-level pass/fail threshold. The preliminary 2.5 m trials informed the choice of the more severe 4.0 m condition because no visually discernible damage was observed at the lower height. However, their quantitative time histories and reproducible damage measurements are not available for this article, so they are excluded from the comparisons and cannot be treated as within-condition replicates. At 4.0 m, the observations describe specimen-specific damage mechanisms, not characteristic impact capacities. Untested candidates, including I30R60, remain unqualified for any mandatory impact requirement regardless of their beam-work or GWP performance. Differences in matrix composition, absent specimen-matched fiber maps, and differences between a supported laboratory slab and a buoyant floating shell further limit transferability. Replicated component testing against predeclared damage, leakage, and residual-capacity criteria is required before using this gate in actual project design.

## 7. Life-Cycle Assessment and Coupled Environmental–Mechanical Performance

### 7.1. Goal, System Boundary, and Inventory

**Figure 17:**
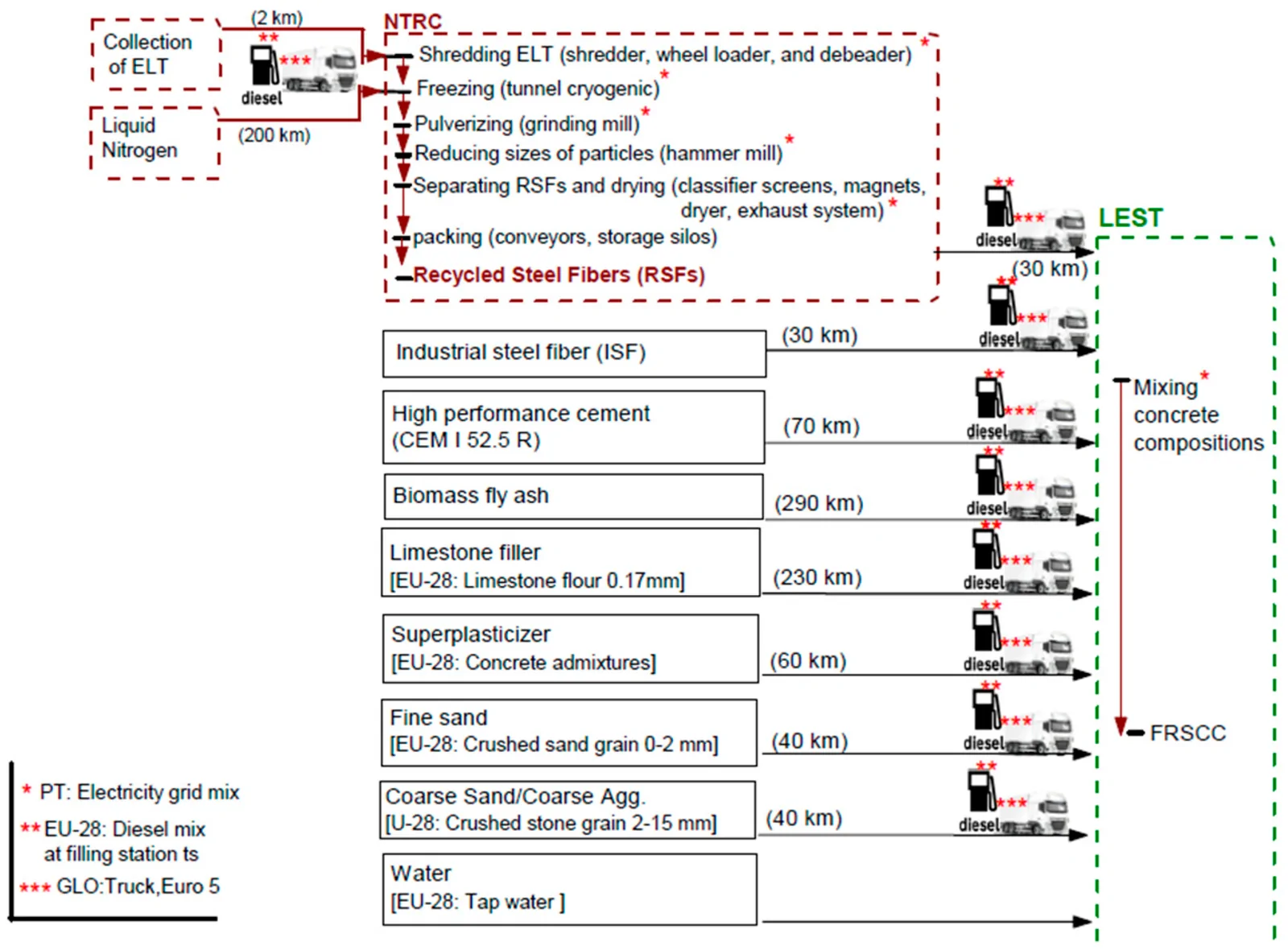
Original cradle-to-gate process system, including fiber recovery, constituent transport, and concrete production.

The source LCA evaluated production of 1 m^3^ of each FRSCC mixture from constituent supply through concrete mixing [2], corresponding to a cradle-to-gate material comparison. The inventory and interpretation are considered within the general framework of ISO 14040 and ISO 14044 [33,34]. Figure 17 retains the original system boundary, including material processing, electricity, and transportation. The core, FRP reinforcement, connectors, installation, maintenance, and end-of-life stages of a complete floating dock are outside that boundary. Accordingly, 1 m^3^ is the declared quantity for the material comparison and does not demonstrate functional equivalence at component or service-life level. The environmental-efficiency concept discussed by Damineli et al. [35] motivates the present performance screen, but normalization by compressive strength alone would neglect the post-cracking mechanisms central to FRC selection.

The inventory combined supplier information, environmental declarations, and generic GaBi datasets (GaBi software (2022 version, Sphera Solutions GmbH, Leinfelden-Echterdingen, Germany). Portuguese electricity and diesel-truck transport were modeled, with approximately 70 km of cement transport and 290 km of fly-ash transport. Fly ash was treated as a waste with no upstream production burden, although transportation was included. The RSF recovery inventory comprised shredding, cryogenic treatment, size reduction, separation, drying, and packing. Reported process inputs were 165.2 kWh of electricity and 750 kg of liquid nitrogen per metric ton of total recovery-plant product, which included rubber, steel fibers, and fluff [2]. These inputs therefore must not be interpreted as consumption per metric ton of isolated RSF.

Transport of end-of-life tires to the municipality was excluded because data were unavailable. The treatment of recovered-material allocation and the cryogenic recovery route are therefore material assumptions in the environmental result. Later work describes a different mechanically recovered fiber supply [3], so the reported cryogenic inventory cannot automatically represent current procurement. The source records also use different terminology for the fly ash, including biomass fly ash and Class F descriptions. Any new project-level LCA should therefore confirm the actual ash source, recovery technology, allocation convention, and supplier-specific transport before adopting the source-study values.

The 2022 LCA recipes also differ from the thesis mixtures in Table 2. For I90R0, the published inventory contained 500 kg cement, 150 kg fly ash, 150 kg filler, 117 kg fine sand, 621 kg coarse sand, and 475 kg coarse aggregate per m^3^, with water and superplasticizer reported as 214 and 17 L [2]. The recycled-only inventory contained 427 kg/m^3^ of coarse aggregate, compared with 472 kg/m^3^ in the thesis mixture. The calculations that follow therefore preserve the published LCA recipes as an internally defined scenario rather than substituting the later thesis proportions.

### 7.2. Environmental Impact Categories

GaBi 6 with the CML 2001 method was used in the source study to quantify global warming potential (GWP), acidification potential (AP), eutrophication potential (EP), fossil-resource abiotic depletion potential (ADP), ozone depletion potential (ODP), and photochemical ozone creation potential (POCP) [2]. These indicators represent different environmental mechanisms and cannot be summed directly in their native units. ISO 14067 [36] specifically addresses carbon footprints; lower GWP therefore cannot be treated as a substitute for the complete environmental profile. Table 7 retains all six impact categories before normalization or weighting.

**Figure 18:**
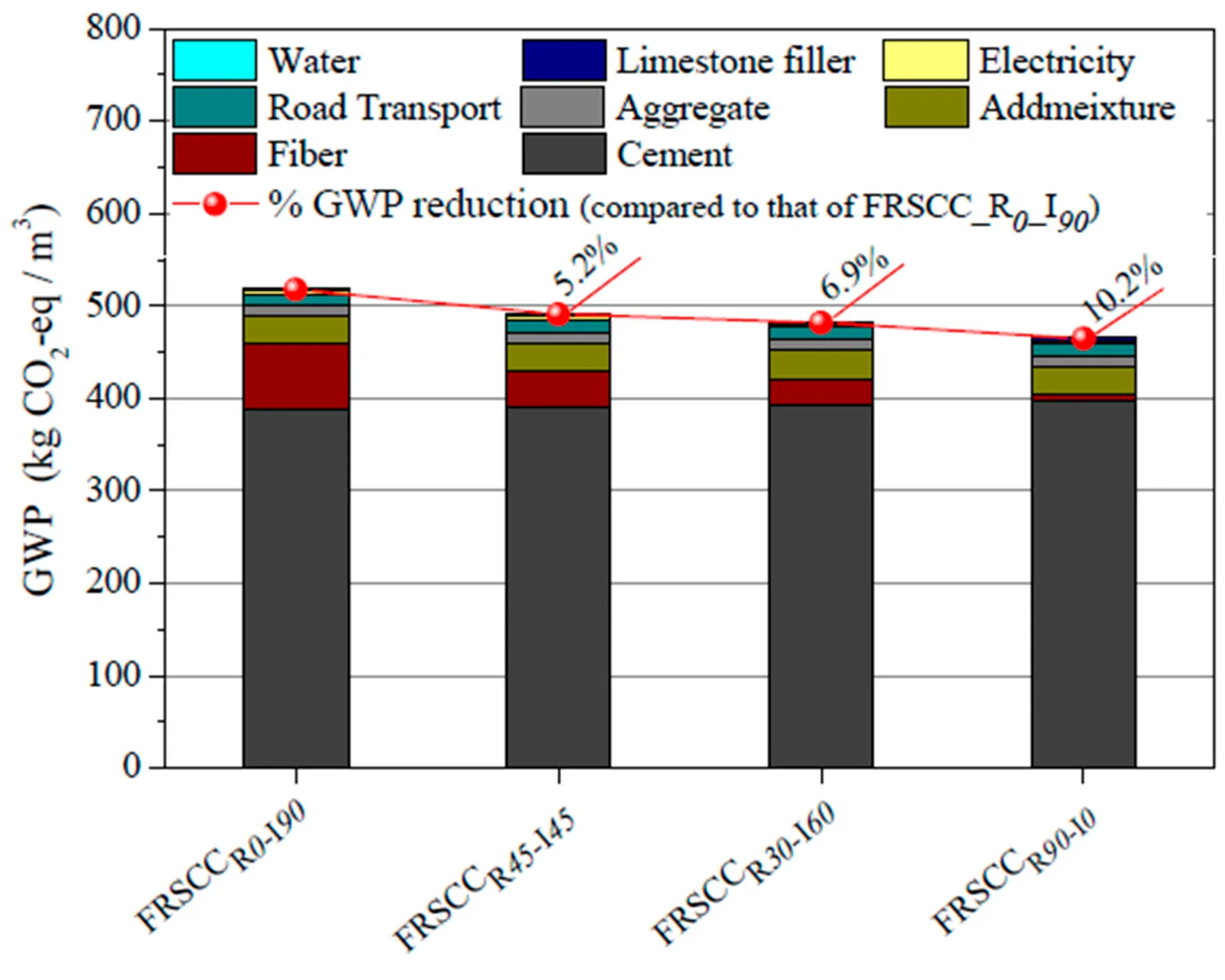
Original contribution analysis of cradle-to-gate GWP.

At equal concrete volume, GWP decreased from 518 kg CO_2_-eq/m^3^ for I90R0 to 491, 482, and 466 kg CO_2_-eq/m^3^ for I45R45, I30R60, and I0R90, corresponding to reductions of approximately 5.2%, 6.9%, and 10.0%. The source attributes approximately 75% of I90R0 GWP to cement and 13.4% to industrial steel fibers. In RSF-containing mixtures, cement contributed approximately 79–85% because fiber-related emissions decreased while matrix demand increased [2]. Figure 18 retains the original contribution analysis. Fiber substitution therefore reduces one environmental burden but does not alter the dominant role of the cementitious matrix.

Relative to I90R0, I0R90 reduced AP by approximately 40%, EP by 44%, and fossil-resource ADP by 47%. Its reported ODP approached a very small value, while POCP became negative within the CML 2001 model used in the source study. The source attributes the negative POCP value to the characterization of transport-related NO emissions [2]. This is a method-specific modeling outcome and must not be interpreted as net removal of atmospheric pollution by concrete production. Very small or negative category values also require particular care during normalization and weighting.

The six-category results demonstrate both the limitations of a carbon-only comparison and the sensitivity of the LCA to inventory assumptions. A current assessment should update electricity, transport, recovery-process, and supplier data; make waste and co-product allocation explicit; and report sensitivity to the dominant assumptions. The published values are deterministic source-study estimates, and no probabilistic environmental uncertainty is inferred from them in the present analysis.

### 7.3. Coupled Environmental–Mechanical Indicators and Their Limitations

**Figure 19:**
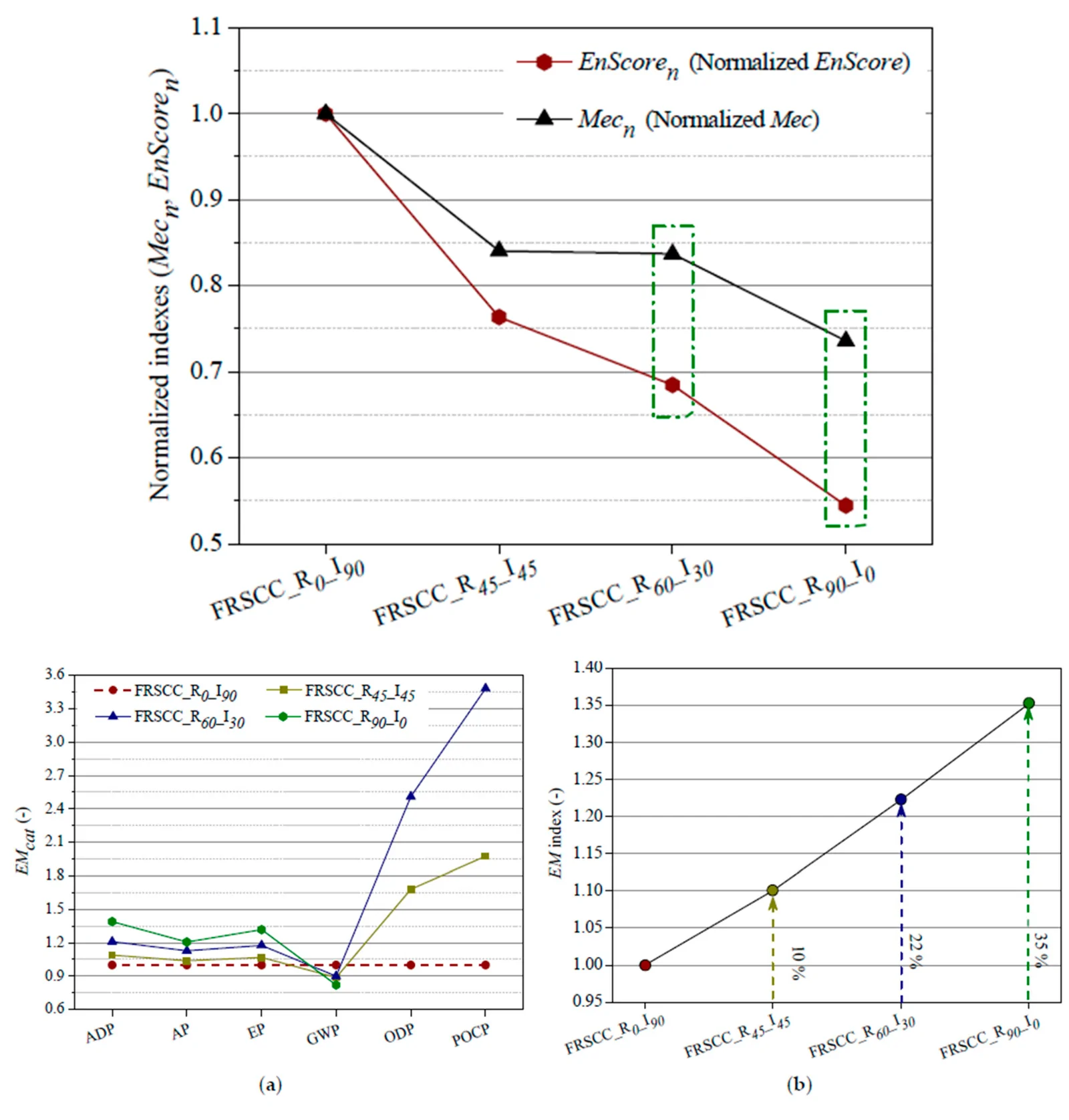
Original normalized mechanical/environmental comparison (**upper panel**) and combined environmental–mechanical evaluation (**lower panels** (**a**,**b**)).

The published environmental score combines normalized and weighted category indicators and takes values of 1.020, 0.779, 0.698, and 0.555 for I90R0, I45R45, I30R60, and I0R90, respectively [2]. The original combined environmental–mechanical index compares normalized beam work with this normalized environmental score. Figure 19 retains that comparison and shows that the weighted environmental score decreases more rapidly than beam work as RSF replacement increases.

Equation (8) reproduces the previous combined index [2] in normalized form: relative beam work (U(i)/U(0)) is divided by the relative weighted environmental score (Senv(i)/Senv(0)). The symbols U and Senv denote beam work and the dimensionless source-study weighted environmental score, respectively; 0 identifies I90R0. The source weighting is retained without adjustment. Recalculation from the rounded published input scores yields relative indices 1.000, 1.101, 1.223, and 1.353. The index is useful as a descriptive benchmark but cannot impose mandatory residual-response thresholds; Equations (9)– (13) add this missing decision capability.

(8) $$\frac{{\mathrm{EM}}_{i}}{{\mathrm{EM}}_{0}}=\frac{\frac{U_{i}}{U_{0}}}{\frac{S_{\mathrm{env},i}}{S_{\mathrm{env},0}}}$$

On this specific aggregate index, I0R90 improves by approximately 35% relative to I90R0. The original abstract reports an improvement of up to 37%; the present value follows directly from the rounded tabulated data. The apparent improvement is therefore conditional on both the chosen environmental denominator and the mechanical response used in the numerator.

An alternative indicator illustrates this dependence. The ratio of normalized GWP to normalized beam work is 1.000, 1.128, 1.112, and 1.222 for I90R0, I45R45, I30R60, and I0R90, respectively; under this carbon-to-work measure, none of the RSF-containing systems outperforms I90R0. The foundational study already separates individual impact categories from its aggregate environmental score [2]. The engineering implication is that the objective function must be stated explicitly and minimum mechanical performance must be imposed before a favorable environmental score is used to justify material selection.

## 8. Integrated Discussion: Performance-Constrained Material Selection for Floating Dock Blocks

### 8.1. Joint Performance Screening, Decision Boundaries, and Candidate Selection

Section 8 presents the methodological advance beyond the single-index assessment of [2]. The earlier assessment compares composite systems using a weighted environmental denominator and one mechanical numerator, so performance losses may be numerically offset by environmental benefits. Here, an explicitly specified requirement must be met for every selected mechanical indicator; environmental preference is considered only within the resulting admissible set. Marine interface and impact properties retain separate pass/fail/untested states, and any unverified mandatory gate prevents final project qualification. Continuous threshold boundaries show when the preferred candidate changes, a sensitivity bound makes close rankings transparent, and carbon-parity calculations measure how much added concrete or detailing burden erases material-level savings. These outputs are derived retrospectively from traceable source data; neither the threshold fractions nor the observed component outcomes represent prescribed marine design limits. Table 8 summarizes the experimental and environmental evidence required for each decision stage, together with the associated limitations and necessary follow-up assessments for floating dock block qualification.

For each mixture i and favorable mechanical indicator k, Equation (9) defines the dimensionless retained response r(i,k) as the ratio of the published mean x(i,k) to the corresponding I90R0 mean x(0,k). The requirement vector q assigns a separate minimum q(k) to each selected indicator in set K; a candidate is mechanically admissible only if every applicable requirement is satisfied. In this demonstration, K comprises beam work U and modified-splitting stresses at 1 mm and 3 mm openings. These are distinct nominal test outcomes and not interchangeable constitutive design strengths. Project-specific interface and impact criteria are assessed independently; missing data cannot be assigned a pass.

(9) $$r_{ik}=\frac{x_{ik}}{x_{0k}},\;A(q)=\{i\;:\;r_{ik}\;\geq \;q_{k}\;\mathrm{for}\;\mathrm{all}\;k\;\in \;K\}$$

Table 9 summarizes the normalized benchmark and joint screening limits. For beam work alone, the retained fractions are approximately 1.000, 0.841, 0.837, and 0.736 for I90R0, I45R45, I30R60, and I0R90. At an illustrative minimum fraction of 0.80, both hybrids satisfy the beam-work criterion, and I30R60 has the lower reported GWP. Adding an independent 0.80 requirement on the 3 mm modified-splitting response excludes I30R60 because its retained fraction is approximately 0.739, whereas I45R45 retains approximately 0.837. On the reported means, the lowest-GWP mechanically admissible candidate therefore changes from I30R60 to I45R45. This is a deterministic case-study result, not a statistically validated population ranking or a marine design limit.

The status columns in Table 9 refer strictly to three mechanical indicators in the paired benchmark. Table 10 translates the subsequent evidence checks into an explicit implementation: an observed non-perforation outcome or reported pull-out curve is documented as “Obs”, but the corresponding project-specific mandatory gate remains “U” until predeclared load, exposure, geometry, failure, and replication criteria have been demonstrated. A mechanical failure ends consideration under the specified q; an untested interface or impact requirement cannot be overridden by lower GWP.

**Figure 20:**
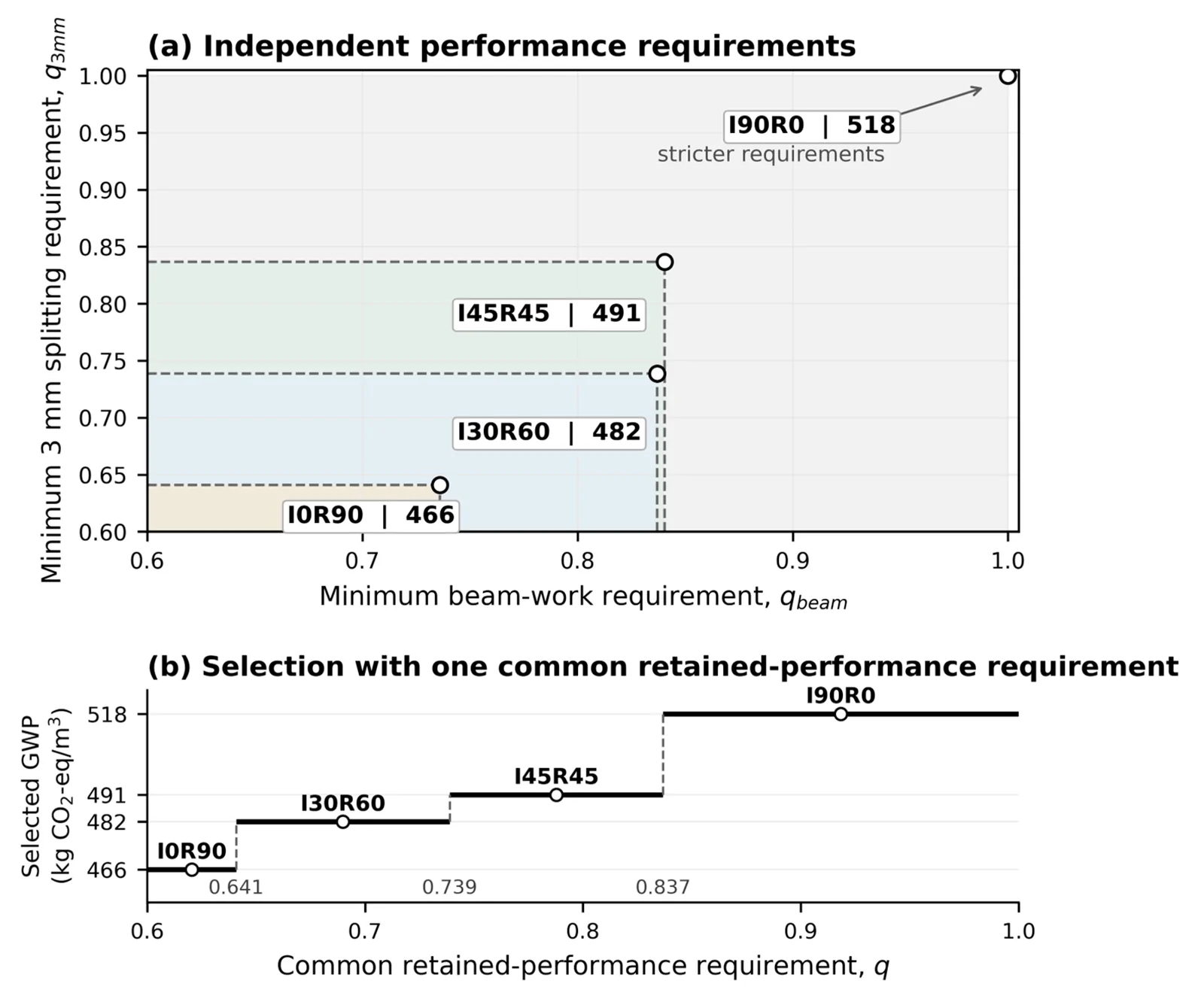
Performance-constrained selection map derived from the reported mean response ratios and GWP values. (**a**) Independent minimum requirements for normalized beam work and the 3 mm modified-splitting response; dashed lines mark the corresponding mixture-specific admissibility limits. (**b**) Stepwise change in the lowest-GWP mechanically admissible candidate when a common retained-performance requirement q is imposed on the screened mechanical indicators. Both panels are deterministic sensitivity representations based on reported means and should not be interpreted as probabilistic design charts.

For illustration only, the same minimum retained fraction q is imposed on beam work and the 1 and 3 mm modified-splitting responses. Equation (10) then identifies the maximum common fraction p(i) supported by each mixture’s reported means. The approximate boundaries are 0.641 for I0R90, 0.739 for I30R60, 0.837 for I45R45, and 1.000 for I90R0. The lowest-GWP mechanically admissible candidate is consequently I0R90 for q ≤ 0.641, I30R60 for 0.641 < q ≤ 0.739, I45R45 for 0.739 < q ≤ 0.837, and I90R0 for 0.837 < q ≤ 1.000. Table 9 states the binary decisions at q = 0.70, 0.80, and 0.90; Figure 20 depicts the continuous boundaries. These are deterministic mean-response selection regions and are not statistical confidence limits.

(10) $$p_{i}=\min\{r_{ik}\;:\;k\;\in \;K\}$$

For the selected mechanical and GWP objectives, all four benchmark mixtures are nondominated: each GWP decrease coincides with loss of at least one mechanical mean, so no unique material optimum exists without declared constraints. The 1 mm response captures an earlier crack-bridging state relevant to crack control, whereas the 3 mm response probes larger-opening bridging and deformation capacity. Neither opening is asserted to correspond directly to an offshore serviceability or ultimate limit state. The 3 mm ratio governs the illustrative common-q boundary for every RSF-containing mixture because it is smaller than their 1 mm and beam-work ratios. In practice, the proper formulation is the vector requirement in Equation (9), with different absolute or normalized limits assigned to beam response and each crack-opening state according to verified structural demand. The common q is used only to visualize sensitivity, not to prescribe identical design acceptance margins for fundamentally different tests.

The close hybrid mechanical means warrant a deterministic ranking-sensitivity test. For two positive reported means a > b, a change that decreases a by η and increases b by η reverses their order once a(1 − η) ≤ b(1 + η). Equation (11) defines the critical equal-opposing perturbation ηcrit = (a − b)/(a + b). This is an algebraic break-even measure, not a standard error, probability of reversal, or experimental confidence interval.

(11) $$\eta_{\mathrm{crit}}=\frac{a-b}{a+b}$$

For the hybrid beam-work means a = 77.967 J and b = 77.637 J, Equation (11) gives ηcrit = 0.212%. For their respective 3 mm stresses, 2.05 and 1.81 MPa give ηcrit = 6.22%. The latter difference is not a statistically proven population ranking: the reported coefficients of variation are 27% and 26% and the direction-specific independent sample counts are unresolved. CoV describes observed dispersion and cannot be used directly as an uncertainty bound on a mean. The baseline GWP ranking of the hybrids is similarly sensitive: equal-opposing shifts of 0.925% in 491 and 482 kg CO_2_-eq/m^3^ lead to parity. No LCA uncertainty distribution is available for a confidence assignment.

The illustrative I45R45 candidate also has a limited margin above q = 0.80: its controlling ratio is 2.05/2.45 = 0.837. Equal opposing fractional perturbations of the candidate and reference 3 mm mean reach the q = 0.80 boundary at approximately 2.24% (obtained by solving [2.05(1 − η)]/[2.45(1 + η)] = 0.80). Its equal-volume GWP advantage over I90R0 disappears under equal opposing shifts of approximately 2.68% in their baseline carbon intensities. These are algebraic break-even changes, not empirical standard errors, confidence intervals, or assigned uncertainty distributions.

Passing the mechanical screen generates a conditional shortlist, not a certified choice. Table 10 distinguishes observed evidence from gate qualification. I45R45 has source conditioned-bond comparisons for specific BFRP/GFRP products and one slab-impact observation; neither dataset defines transferable acceptance thresholds for the proposed shell. I90R0 and I0R90 have one impact observation each but no comparable conditioned-bond qualification in the integrated evidence, while I30R60 has neither a matching impact slab nor matching conditioned-bond data. Thus, an untested mandatory gate is explicitly unresolved and no studied mixture can be designated fully project-qualified on these records. New tests must employ the actual geometry, exposure, impact demand, acceptance criteria, and independent replication.

The ranking reversal between I30R60 and I45R45 is consistent with the broader recycled-fiber literature in one important respect: performance is governed by fiber geometry, anchorage, hybridization, and casting-induced orientation rather than recycled origin alone. The present analysis extends that observation from a material-comparison problem to a decision problem. A mixture can be preferable at one crack-opening state and inadmissible at another, and a lower material-level GWP cannot compensate for an unverified bond interface or component impact response. This non-compensatory treatment is particularly relevant to marine shells, where durability and damage containment are functional requirements rather than optional attributes of an eco-efficiency index.

### 8.2. Detailing-Sensitive Carbon Budgets

A 1.20 × 1.20 × 0.10 m panel, matching the tested slab dimensions, has a concrete volume of 0.144 m^3^; at 90 kg/m^3^ total steel fibers, this corresponds to 12.96 kg of fibers per panel. Applying the 2022 source inventory to this common volume yields the illustrative concrete-production burdens summarized in Table 11. These are newly calculated scenario values rather than verified footprints of the later impact slabs. Their purpose is to quantify how subsequent detailing can consume an apparent material-level carbon advantage without assuming structural equivalence among the mixtures.

For an alternative requiring concrete volume Vi and additional non-concrete burden ΔCi (e.g., changed reinforcement, transport or detailing), Equation (12) states the carbon condition for improving on a reference panel of volume V0 and intensity c0. Here, ci is the candidate concrete GWP in kg CO_2_-eq/m^3^, Vi and V0 are in m^3^, and ΔCi is in kg CO_2_-eq; 0 denotes I90R0. All terms require the same declared life-cycle boundary and should be evaluated consistently for both alternatives. Added concrete is already represented by Vi and must not be double counted in ΔCi.

(12) $$c_{i}V_{i}+{\Delta C}_{i}<c_{0}V_{0}$$

Equation (13) provides the maximum allowable additional concrete volume as a fraction of V0 at carbon parity: ΔVi,max = Vi,max − V0. Its denominator ciV0 represents the candidate’s base-panel concrete burden. If the right-hand side is negative, an additional non-concrete burden has consumed the baseline saving before any added concrete is allowed.

(13) $$\frac{{\Delta V}_{i,\max}}{V_{0}}=\frac{c_{0}}{c_{i}}-1-\frac{{\Delta C}_{i}}{c_{i}V_{0}}$$

With all other burdens unchanged, the carbon-parity volume allowances are 5.50%, 7.47%, and 11.16% for I45R45, I30R60, and I0R90, respectively. For a 100 mm panel, uniform thickness increases of only 5.50, 7.47, and 11.16 mm would consume those savings. These are environmental accounting limits and not structurally permissible thickness changes. Under the illustrative joint 0.80 screen, selecting I45R45 rather than I30R60 increases GWP by 1.30 kg CO_2_-eq per panel (1.87%) while satisfying the additional late-crack requirement on the reported mean values.

Local thickening can be evaluated without adopting an unverified anchorage model. For local plan area Aadd and thickness increment Δt, the incremental concrete burden is ci Aadd Δt, with Aadd in m^2^ and Δt in m. With an illustrative 50 mm increment and no additional non-concrete burden, the carbon-parity areas are 0.158, 0.215, and 0.321 m^2^ for I45R45, I30R60, and I0R90, respectively (Table 11). The I45R45 allowance is 11.0% of the 1.44 m^2^ panel area. Changed reinforcement or additional processing reduces the allowance; overlapping thickened areas are counted only once. These limits quantify environmental parity, not structural adequacy.

This accounting directly informs the anchorage-transfer problem. Reproducing the tested cube’s nominal cover on both faces of a centered 12 mm bar would require an overall thickness of 150 mm, although equal cover alone would still not reproduce the cube confinement. Uniformly increasing the I45R45 panel from 100 to 150 mm would raise its concrete-production burden to 106.06 kg CO_2_-eq, approximately 42% above the 100 mm I90R0 reference. Local thickening is therefore a hypothesis for design development, not a prequalified solution; adequate anchorage, confinement, and post-impact watertightness must be verified independently.

The 1.30 kg CO_2_-eq difference between the two hybrids is particularly sensitive to detailing. At the I30R60 carbon intensity, this saving is exhausted by only 2.69 L of additional concrete, equivalent to a uniform thickness increase of 1.87 mm over the panel. Material-level GWP ranking can therefore be reversed by very small quantity changes, directly linking crack-opening requirements, material selection, and detail design.

A differential, rather than common-mode, inventory perturbation is decisive for the two hybrids. From the rounded source intensities c(I45R45) = 491 and c(I30R60) = 482 kg CO_2_-eq/m^3^, their baseline difference is 9 kg CO_2_-eq/m^3^, or 1.296 kg CO_2_-eq for a 0.144 m^3^ panel. If both intensities receive equal and opposing relative changes η, carbon parity occurs at η = (491 − 482)/(491 + 482) = 0.925%. For example, decreasing I45R45 by 1% and increasing I30R60 by 1% changes their values to 486.09 and 486.82 kg CO_2_-eq/m^3^, reversing the baseline ranking. In contrast, an equal 5% change in both inventories preserves their order because correlated shifts do not erase the difference. This is a scenario-based break-even analysis, not an empirical uncertainty interval or a claim that ±1% represents a measured LCA error.

The sensitivity can also be stated in inventory units without inventing process emission factors. A differential shift of 9 kg CO_2_-eq/m^3^ (equivalently 1.296 kg CO_2_-eq per panel) is sufficient for parity if applied to only one mixture. For example, if attributed entirely to the extra 15 kg/m^3^ of recycled rather than industrial fiber in I30R60 compared with I45R45, the break-even differential attribution would be 0.60 kg CO_2_-eq/kg of substituted fiber; this is a mathematical equivalent, not a measured RSF factor. Differences in fiber-recovery allocation, transport, electricity source, and the nonidentical mixture recipes may act together and may be correlated; without unit-process contributions and plausible supplier-specific ranges, the relative effect of each cannot be resolved. Consequently, the relative GWP preference of the hybrids is not robust to small differential changes, whereas the absolute mechanical admissibility condition at q = 0.80 continues to exclude I30R60 irrespective of its environmental score. A prospective LCA should report alternative recovery/allocation cases, transport distances, and electricity scenarios before procurement decisions.

### 8.3. Generalizability, Limitations, and Verification Priorities

The framework is transferable in logic, but its numerical boundaries and mixture rankings remain specific to the investigated sources. The experimental programs did not always use identical matrix formulations or concrete batches; numerical integration is therefore restricted to the internally paired benchmark. Inconsistent reporting of modified-splitting specimen counts and geometry means that the 1 and 3 mm stresses are descriptive published means rather than characteristic design strengths. The quantitative impact evidence comprises one documented slab per mixture at 4.0 m, with I30R60 untested. Exploratory 2.5 m impacts, which showed no visually discernible damage and motivated the higher test level, cannot be included in the response comparisons because their quantitative records are not available for consistent re-analysis. Nor do trials at different impact heights constitute replicates at a common loading level. In addition, the raw synchronized signals and complete signal-processing metadata are unavailable for independent high-frequency verification. The impact gate is consequently a proof-of-concept demonstration of the decision logic, not a statistically established acceptance test. The cradle-to-gate LCA is deterministic and does not establish supply-chain uncertainty or service-life equivalence. Complete-dock buoyancy, watertightness, global stability, connections, mooring, fatigue, and long-term durability remain unverified. DNV-ST-C502 [37] provides relevant offshore-concrete design and durability context but does not establish compliance.

These limitations define a prospective validation program. First, direction-dependent residual mechanical response should be tested on independently produced, representative castings, with documented specimen counts, geometry, and corresponding fiber fields. Conditioned FRP-interface tests should reproduce the intended reinforcement product, cover, confinement, and marine exposure. At component scale, replicated impacts at each specified energy level should employ calibrated and synchronized instrumentation, predefined functional acceptance criteria, and post-impact leakage and residual-capacity measurements. A separate future investigation may compare 2.5 and 4.0 m responses once the lower-height records have been retrieved, documented, and assessed on a consistent basis; this future comparison is not a result of the present study. Finally, the LCA should be updated for the qualified production recipes and component details using justified scenarios for recovery allocation, electricity, and transport. Independent demonstrator validation is essential. This framework is intended to support preliminary material screening and to organize the subsequent verification work relevant to DNV-ST-C502, not to replace standards-based design or certification.

## 9. Conclusions

This study proposes and illustrates a non-compensatory, performance-constrained screening method for selecting FRSCC for floating dock blocks. Its original outputs are the joint admissibility formulation, continuous material-selection boundaries, deterministic ranking-sensitivity limits, and detailing-sensitive component carbon budgets. Environmental preference is evaluated only after the specified mechanical responses are satisfied, while interface and component evidence remain separate gates. The method therefore does not identify a universally optimal fiber blend or claim final project qualification; it identifies the lowest-reported-burden candidate that remains mechanically admissible for an explicitly stated set of requirements and detailing assumptions, subject to verification of the remaining mandatory gates.

At the material scale, compressive strength and elastic modulus were relatively insensitive to fiber replacement (approximately 60–66 MPa and 30–31 GPa), whereas post-cracking response changed substantially. Direction-dependent modified splitting together with DIA and micro-CT further showed that neither fiber mass dosage nor planar intersection count is a sufficient proxy for crack-bridging capacity when orientation, anchorage, and morphology differ.

At the interface and component scales, the 24-month conditioned BFRP groups exhibited mean peak bond stresses of approximately 44–51% of the 28-day control values, whereas the tested GFRP product showed approximately 96–107%, [38]. The simultaneous differences in age and conditioning prevent these ratios from being interpreted as isolated seawater effects, and product-specific slip and failure mechanisms remain important. In the four documented 4.0 m slab tests (nominal incident energy 4.12 kJ), the three tested FRSCC slabs did not perforate. Earlier exploratory impacts at approximately 2.5 m produced no visually discernible damage, which motivated the higher test level; the associated quantitative records are not included in this study and no cross-height performance ranking is claimed. Because only one slab per composition was tested at 4.0 m and I30R60 was not tested, the impact observations demonstrate only the proof-of-concept logic of the non-compensatory component gate, not a statistically defensible design pass/fail rule. Replicated component testing, traceable signal processing, and post-impact residual-capacity and watertightness assessment are required for qualification. Comparative analysis across different drop heights is reserved for future work after the lower-height measurements have been documented and verified.

The joint screen shows that material preference changes with the required crack-opening state. For a common retained-performance requirement q, the lowest-reported-GWP mechanically admissible candidate progresses from I0R90 to I30R60, I45R45, and I90R0 as q crosses approximately 0.641, 0.739, and 0.837, respectively. At the illustrative q = 0.80-point, beam work alone admits I30R60, whereas adding the 3 mm modified-splitting requirement admits I45R45 as the lower-GWP hybrid satisfying both conditions. The two-constraint decision map confirms that this is a boundary effect rather than a result tied to one arbitrary threshold. Because the hybrid mechanical and GWP differences are narrow and the source datasets are limited, uncertainty characterization and prospective validation remain necessary before production-scale selection.

For the 0.144 m^3^ reference panel, I45R45 has a 3.89 kg CO_2_-eq cradle-to-gate advantage over I90R0, corresponding to only 5.50% additional concrete before carbon parity. The 1.30 kg CO_2_-eq advantage of I30R60 over I45R45 is consumed by 2.69 L of additional I30R60 concrete. These new derivative quantities demonstrate the cross-scale engineering consequence of the framework: a lower GWP at equal material volume is advantageous only if the selected material also satisfies the required post-cracking response and does not require interface or component detailing that consumes the environmental margin.

The environmental ranking between I45R45 and I30R60 is sensitive to less than 1% equal-opposing changes in their reported GWP intensities, so supply-chain transport, recycled-fiber allocation and processing choices must be revisited before interpreting a 1.30 kg CO_2_-eq panel difference as decisive. The resulting framework is best used to pre-screen materials and define the subsequent component, interface, and durability verification program in the context of standards such as DNV-ST-C502 [37], not as proof that any mixture or floating dock already meets the standard.

## Figures and Tables

**Table 1:** Evidence provenance, compatibility, and original analytical use in the present study.

Program	Evidence and Provenance	Compatibility and New Use in This Study
Constituents and fresh concrete	Constituent characterization, mixing, slump flow, and fiber morphology reported in [1,2,3,4].	Compatibility: Contextual/production evidence; program-specific recipes are not pooled. New use: Defines material identity, manufacturing constraints, and production evidence required before transfer to components.
Cylinder and beam mechanics	Four fiber systems; three cylinders/beams per mixture in the paired dataset [1,2].	Compatibility: Direct numerical input where values belong to the paired benchmark family. New use: Provides normalized beam-work response for Equations (9)–(11) and the continuous decision boundaries.
Modified splitting, DIA, and CT	Directional/depth splitting and fiber-field characterization; one CT specimen per mixture [1,2,3].	Compatibility: Splitting means from the paired family enter screening; DIA/CT remain diagnostic evidence. New use: Provides crack-opening constraints and demonstrates why dosage/count cannot substitute for casting-sensitive post-cracking screening/qualification.
Marine FRP bond	Twenty-four cubes; BFRP/GFRP; static/post-cycle; 28-day controls and 24-month conditioned groups [4].	Compatibility: Transfer-relevant interface evidence; not merged numerically into the mechanical/LCA ranking. New use: Acts as an independent interface gate and identifies cover, confinement, age/exposure, and product-specific verification requirements.
Component impact	Four documented 4.0 m slab tests (one per tested composition) at 28 days, using a 105 kg projectile [1,5]; earlier exploratory 2.5 m trials are not included in the quantitative dataset.	Compatibility: Specimen-level 4.0 m observations only; no population-level ranking and no batch-specific transfer of LCA. New use: Proof-of-concept component gate based on observed damage, contact force, acceleration, and deformation; untested candidates remain unqualified.
Life-cycle assessment	Four paired recipe scenarios; declared quantity 1 m^3^; six CML categories [2].	Compatibility: Direct environmental input only within the paired benchmark family. New use: Provides GWP for admissible-candidate selection and the component carbon-parity analysis.

**Table 2:** Mixture proportions for the four fiber-reinforced concrete systems and the plain impact mixture. All values are kg/m^3^.

Constituent	I90R0	I45R45	I30R60	I0R90	Plain
Cement	472	504	506	511	506
Fly ash	141.5	200	201	203	201
Limestone filler	142	151.1	152	153	152
Fine sand	123	107	113	119	113
Coarse sand	556	568	552.5	535	552.5
Coarse aggregate	503	435	432	472	523
Water	201.7	231.34	232.6	234.7	232.6
Superplasticizer	15.11	17.1	17.2	17.34	17.2
ISF	90	45	30	0	0
RSF	0	45	60	90	0

**Table 3:** Reported 28-day cylinder properties and beam work. Parentheses give coefficients of variation (%); no uncertainty estimate for beam work is supplied.

Mixture	Compression (MPa)	Modulus (GPa)	Beam Work (J)
I90R0	66.10 (1.31)	31.41 (1.15)	92.762
I45R45	63.45 (1.03)	30.33 (1.22)	77.967
I30R60	63.12 (1.27)	30.04 (1.15)	77.637
I0R90	60.03 (1.29)	30.00 (1.31)	68.276

**Table 4:** Modified-splitting mean values with coefficients of variation (%) in parentheses. σ is nominal stress; G is the integral of nominal stress against opening, not beam work.

Indicator (Unit)	I90R0	I45R45	I30R60	I0R90
$\sigma_{\max}$ (MPa)	5.63 (19)	4.78 (16)	4.54 (13)	3.73 (23)
$\sigma_{0.3}$ (MPa)	5.58 (21)	4.74 (15)	4.54 (16)	3.70 (25)
$\sigma_{0.5}$ (MPa)	5.01 (24)	4.51 (16)	4.32 (16)	3.57 (27)
$\sigma_{1.0}$ (MPa)	3.94 (25)	3.77 (14)	3.50 (22)	3.06 (27)
$\sigma_{1.5}$ (MPa)	3.44 (28)	3.18 (14)	2.89 (26)	2.48 (26)
$\sigma_{3.0}$ (MPa)	2.45 (30)	2.05 (27)	1.81 (26)	1.57 (25)
$G_{0.3}$ (N/mm)	1.49 (17)	1.29 (14)	1.26 (14)	1.03 (22)
$G_{0.5}$ (N/mm)	2.54 (19)	2.21 (15)	2.15 (14)	1.75 (24)
$G_{1.0}$ (N/mm)	4.74 (21)	4.26 (14)	4.10 (15)	3.40 (26)
$G_{1.5}$ (N/mm)	6.58 (22)	5.98 (14)	5.68 (18)	4.78 (26)
$G_{3.0}$ (N/mm)	10.84 (30)	9.80 (15)	9.10 (20)	7.78 (24)

**Table 5:** Bond response and specimen failure modes. Each group contains three specimens.

Group	Peak τ (MPa)	Slip at Peak (mm)	τ at 0.05 mm (MPa)	Conditioned/Control Peak Ratio (%)	Failures: Splitting/Slip
B-D-S	27.39	2.06	8.61	—	1/2
B-W-S	12.14	3.80	4.00	44.32	0/3
B-D-F	27.30	2.94	6.50	—	3/0
B-W-F	13.90	3.24	4.60	50.92	0/3
G-D-S	23.50	0.50	6.50	—	0/3
G-W-S	22.46	0.98	12.74	95.57	1/2
G-D-F	24.16	1.62	1.40	—	2/1
G-W-F	25.78	1.50	11.15	106.71	2/1

**Table 6:** Reported observations from the documented 4.0 m impact tests (one slab per tested composition). Deflection ranges are approximate readings; acceleration values are magnitudes of dominant negative peaks. The exploratory 2.5 m trials are not included.

Response	Plain	I90R0	I45R45	I0R90
Perforation/rear scabbing	Yes/yes	No/no	No/no	No/no
Peak average deflection (mm)	Not comparable	38–39	42–43	39–40
Terminal average deflection (mm)	Not comparable	3.5–4.0	5–6	13–14
Peak contact force (kN)	110.00	285.83	256.52	258.00
Negative acceleration magnitude (g)	296.79	780.26	710.25	552.62
Maximum crack width (mm)	Not reported	10	9	9
Bottom damaged-zone diameter (cm)	54.00	28.27	38.77	30.41
Bottom damaged-zone area (cm^2^)	1068.24	530.00	369.38	237.27

**Table 7:** Cradle-to-gate environmental impacts per m^3^ and environmental score.

Category (Unit)	I90R0	I45R45	I30R60	I0R90
GWP (kg CO_2_-eq)	518	491	482	466
ODP (kg R11-eq)	0.009	0.004	0.003	4.53 × 10^−9^
AP (kg SO_2_-eq)	0.210	0.170	0.156	0.127
EP (kg PO_4_-eq)	0.0613	0.0485	0.0437	0.0343
POCP (kg C_2_H_4_-eq)	0.026	0.011	0.006	−0.004
Fossil ADP (MJ-eq)	1720	1330	1190	912
Environmental score (–)	1.020	0.779	0.698	0.555

**Table 8:** Proposed use of experimental and environmental evidence.

Decision	Evidence to Use	Transfer Limit/Required Follow-Up
Production	Constituent tests; fresh properties; DIA/CT	Qualify actual mold, flow path, fiber lot and local variability.
Mechanical resistance	Compression, modulus, residual flexure and splitting	Derive appropriate design quantities; do not exchange nominal test stresses.
Anchorage and durability	Conditioned static/post-cycle bond; failure observations	Test selected bar, cover, confinement and relevant exposure/loading.
Accidental impact	Damage; force; acceleration; peak/terminal deflection	Replicate component tests; measure leakage and residual capacity.
Environmental preference	Six-category LCA of compatible material recipes	Screen required performance first; update inventory and detail quantities.
Floating-unit qualification	Complete geometry, mass and interface information	Verify buoyancy, stability, joints, mooring, core and damaged function.

**Table 9:** Mechanical screening using reported mean response ratios. “Admiss.” and “Inadmiss.” denote mechanical admissibility at the stated common q, not full project qualification. Subscripts U, 1, and 3 refer to beam work and 1 and 3 mm splitting stresses, respectively.

q = 0.90	q = 0.80	q = 0.70	*p* = min(r)	r3	r1	rU	GWP (kg CO_2_-eq/m^3^)	Mixture
Admiss.	Admiss.	Admiss.	1.000	1.000	1.000	1.000	518	I90R0
Inadmiss.	Admiss.	Admiss.	0.837	0.837	0.957	0.841	491	I45R45
Inadmiss.	Inadmiss.	Admiss.	0.739	0.739	0.888	0.837	482	I30R60
Inadmiss.	Inadmiss.	Inadmiss.	0.641	0.641	0.777	0.736	466	I0R90

**Table 10:** Evidence-status matrix for the illustrative q = 0.80 screen. P = meets the nominal mean-response mechanical screen; F = fails; U = untested or unqualified against project criteria; Obs = single specimen/experimental comparison available, without validated acceptance.

Overall Project Status	Slab Impact	Conditioned FRP Interface	Mechanics (q = 0.80)	Mixture
U	Obs; gate U	U (no matching test)	P	I90R0
U	Obs; gate U	Obs (specific bars); gate U	P	I45R45
F	U (no slab)	U	F	I30R60
F	Obs; gate U	U (no matching test)	F	I0R90

**Table 11:** Component carbon accounting for a 0.144 m^3^ panel. Additional-volume and local-area limits assume unchanged burdens elsewhere. Local areas assume 50 mm additional thickness.

Mixture	Concrete GWP (kg CO_2_-eq)	Saving (kg CO_2_-eq)	Extra Volume at Parity (%)	Local Area at Parity (m^2^)
I90R0	74.59	0.00	0.00	0.000
I45R45	70.70	3.89	5.50	0.158
I30R60	69.41	5.18	7.47	0.215
I0R90	67.10	7.49	11.16	0.321

## Data Availability

The original contributions presented in this study are included in the article. Further inquiries can be directed to the corresponding author.
